# Machine learning based modeling of households: A regionalized bottom‐up approach to investigate consumption‐induced environmental impacts

**DOI:** 10.1111/jiec.12969

**Published:** 2019-11-24

**Authors:** Andreas Froemelt, René Buffat, Stefanie Hellweg

**Affiliations:** 1 Chair of Ecological Systems Design, Institute of Environmental Engineering, ETH Zurich, Zurich, Switzerland; 2 Chair of Geoinformation Engineering, Institute of Cartography and Geoinformation, ETH Zurich, Zurich, Switzerland; 3 Institute of Environmental Engineering, Ecological Systems Design, ETH Zurich, HPZ E32.2, John‐von‐Neumann‐Weg 9, Zurich, CH‐8093 Switzerland

**Keywords:** data mining, household consumption, industrial ecology, large‐scale bottom‐up model, machine learning, spatial analysis

## Abstract

As major drivers of economy, households induce a large share of worldwide environmental impacts. The variability of local consumption patterns and associated environmental impacts needs to be quantified as an important starting point to devise targeted measures aimed at reducing household environmental footprints. The goal of this article is the development and appraisal of a comprehensive regionalized bottom‐up model that assesses realistic environmental profiles for individual households in a specific region. For this purpose, a physically based building energy model, the results of an agent‐based transport simulation, and a data‐driven household consumption model were interlinked within a new probability‐based classification framework and applied to the case of Switzerland. The resulting model predicts the demands in about 400 different consumption areas for each Swiss household by considering its particular circumstances and produces a realistic picture of variability in household environmental footprints. An analysis of the model results on a municipal level reveals per‐capita income, population density, buildings' age, and household structure as possible drivers of municipal carbon footprints. While higher‐emission municipalities are located in rural areas and tend to show higher shares of older buildings, lower‐emission communities have larger proportions of families and can be found in highly populated regions by trend. However, the opposing effects of various variables observed in this analysis confirm the importance of a model that is able to capture regional distinctions. The overall model constitutes a comprehensive information base supporting policymakers in understanding consumption patterns in their region and deriving environmental strategies tailored to their specific population.

**Supplementary Information:**

The online version of this article (doi:10.1111/jiec.12969) contains supplementary material, which is available to authorized users.

## INTRODUCTION

Households initiate a multitude of economic activities along the supply chains of consumed products and services, most of which involving the use of resources and the release of emissions. Ivanova et al. (2016) estimate the households' shares to be 65% of global greenhouse gas (GHG) emissions and 50–80% of total land, material, and water use. In view of anthropogenic impacts exceeding the carrying capacity of the earth system (Steffen et al., 2015), deep changes in today's consumer behaviors are urgently needed (e.g., Hertwich, 2005; Minx et al., 2009; Schanes, Giljum, & Hertwich, 2016; Tukker, Cohen, Hubacek, & Mont, 2010). However, several factors such as economic constraints, breaking existing socio‐cultural systems, and complicated supply chains make it difficult for individual households to change their lifestyles and reduce their environmental footprints (O'Rourke & Lollo, 2015).

Regardless of the ongoing debate on whom responsibilities fall (Afionis, Sakai, Scott, Barrett, & Gouldson, 2017), policymakers may assume a key role in shaping an environment that enables shifts toward more sustainable forms of consumption (O'Rourke & Lollo, 2015; Schanes et al., 2016; Tukker et al., 2010) by introducing, for instance, financial incentives, regulations, best‐practice guidance, or extending infrastructure. Initiatives of local authorities on a municipal, regional, or city level are increasingly acknowledged as playing a substantial role in this regard (Moran et al., 2018; UN‐HABITAT, 2011), particularly because they are close to the wants and needs of local actors.

An essential prerequisite to successfully identify targeted measures and effective sets of policy instruments is quantitative information on local households and the environmental consequences of their consumption patterns (Horta & Keirstead, 2017). Many existing environmental assessments apply top‐down approaches and focus on national average households (e.g., Bin & Dowlatabadi, 2005; Hertwich, 2011; Hertwich & Peters, 2009; Ivanova et al., 2016; Tukker, Eder, & Suh, 2006) or investigate different household groups within a certain country (e.g., Girod & De Haan, 2009, 2010; Jones & Kammen, 2011; Weber & Matthews, 2008). While these studies are appropriate to conduct international comparisons and to provide important insights into environmental hotspots of consumption and general tendencies at a national scale, they are too coarse for deriving targeted measures tailored to the problems of a particular region (Horta & Keirstead, 2017; Moran et al., 2018). The complexity of household consumption and the variability of lifestyles clearly indicate that “one‐size‐fits‐all” solutions are likely to fail and thus more detailed information in a high spatial resolution is required for effective policymaking (Jones & Kammen, 2011; Lenzen et al., 2006; Minx et al., 2009).

Collecting adequate data to capture the diversity of consumption behaviors in a specific area is usually too cumbersome and thus beyond the scope of local authorities' capabilities (Froemelt, Mauchle, Steubing, & Hellweg, 2018). To avoid laborious data gathering and hence to reduce this barrier to act, models can represent valuable means to tailor information from existing databases to local conditions.

Few regionalized models exist that map and disaggregate the total environmental footprint of the study area to spatial sub‐scales (e.g., Baiocchi, Minx, & Hubacek, 2010; Druckman & Jackson, 2008; Jones & Kammen, 2014; Minx et al., 2013; Moran et al., 2018). By focusing on the assessment of the average household of smaller sub‐areas, these approaches might account for a certain spatial variation but simultaneously remain on aggregated levels to some extent. However, to develop effective measures that are tailored to the actual inhabitants of a region, even more specific knowledge about the households and the consequences of their respective lifestyles is required. Capturing and understanding the variability of local consumption patterns is particularly important for—but not limited to—soft policy instruments (e.g., awareness raising campaigns, personalized messages) whose design should consider aspects of behavioral economics or psychology (Frederiks, Stenner, & Hobman, 2015; O'Rourke & Lollo, 2015). Since purchase decisions are made on a household level, a bottom‐up approach that takes individual households as the central elements of modeling would be most desirable to explicitly model the variability of household behavior within the study region.

Modeling individual households provides not only the possibility to generate highly regionalized data, but also produces a model with the flexibility to aggregate results on different spatial scales, meaning policymakers can be informed on various levels (e.g., municipal, district, or national levels). Saner et al. (2016), Saner, Heeren, Jäggi, Waraich, and Hellweg (2013) developed promising household‐centered modeling approaches. Though their ideas inspired the basis for the current article, their models neither cover all consumption areas nor do they preserve the context of total household consumption because the categories mobility, food, and housing were modeled independent of each other. Furthermore, these models have never been applied on large scale.

Moreover, currently, there is an ever increasing amount of available data, which has resulted in the term “Big Data” (Xu, Cai, & Liang, 2015). However, in the context of providing information for policymakers, not only the sheer mass of available data is interesting, but especially the new approaches to exploit these datasets. These so‐called data mining or machine learning techniques have been successfully applied to identify and interpret patterns in data and to predict unknown variables (Kuhn & Johnson, 2013; North, 2012; Xu et al., 2015). Although the large potential of the machine learning toolbox for the discipline of industrial ecology has been recognized (Xu et al., 2015), few studies in this field have already employed these tools (e.g., Cai & Xu, 2013; Li, Zhang, Du, & Liu, 2017; Sundaravaradan, Marwah, Shah, & Ramakrishnan, 2011; Wernet, Hellweg, Fischer, Papadokonstantakis, & Hungerbuhler, 2008) and—to our knowledge—no study exists that deploys machine learning techniques in the context of household‐induced environmental impacts. However, in view of the complex domain of household consumption behavior, the use of techniques that are designed to deal with complexity, multidimensionality, and nonlinearities (Kuhn & Johnson, 2013) seem to be able to provide promising contributions to this field of study.

Against the background of above identified requirements, the underlying goal of this article is the development and evaluation of a spatially resolved bottom‐up household consumption model that is able to predict a realistic environmental profile for each household in a region. This model shall provide a comprehensive information base by quantifying the variability of environmental impacts induced by individual households. Although some of the aforementioned spatially resolved models work with micro‐data, consider different household types or integrate data from household characteristics, none of these pursue such a household‐centered approach and provide estimates of total consumption and associated impacts for individual households. This becomes more obvious in a recent review on consumption‐based carbon footprint studies in which the reviewed research is organized according to its spatial dimension (international, national, sub‐national, city, and sub‐city) (Ottelin et al., 2019). The authors, however, explicitly exclude the household level due to its lack of a geographic reference. To our knowledge, our article thus presents a first attempt to estimate consumption behavior and associated environmental impacts for individual households on large scale, and hence provides quantitative information facilitating analyses from the household level up to the national level. Furthermore, we will employ machine learning techniques that will allow us to exploit existing databases and to preserve the context of total household consumption. We believe that this new level of detailed information provides improved support for policymakers in identifying target groups of consumers and in deriving effective measures aimed at the reduction of household‐induced environmental impacts. In addition to providing detailed data, the presented model is designed to evaluate scenarios of planned policies.

In brief, the main goal of our article is the presentation of a new approach for modeling individual households in an unprecedented level of detail. Further objectives comprise: (a) showcasing how machine learning techniques can be deployed in household consumption studies; (b) the application of our approaches to the case of Switzerland, in order to demonstrate the feasibility of operating our modeling framework on a large scale; (c) a status quo analysis of household carbon footprints aggregated on municipal level; and (d) a hectare‐based investigation of linkages between population density and GHG emissions. The latter two analyses mostly aim at providing a glimpse of the model's capabilities, while the computation of detailed scenarios is left for upcoming research.

## METHODS

### Overview of the modeling approach and system boundaries

**Figure 1:**
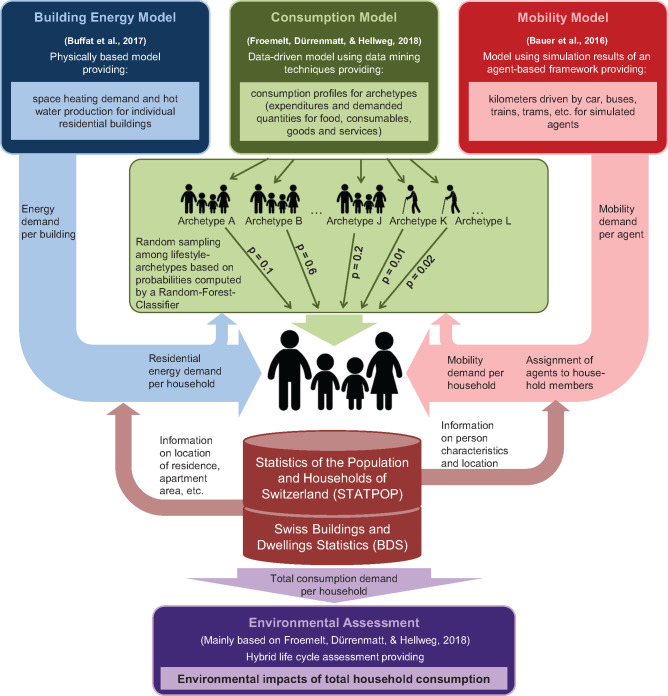
Simplified flow scheme of the modeling framework. The results of the building energy sub‐model and the mobility sub‐model are used in the computation of the probabilities for the assignment of the consumption sub‐model's archetypes to households in order to interlink the three sub‐models

In order to develop such a model platform, three sub‐models were linked (see Figure 1): a physically based building energy model, a data‐driven consumption model, and a mobility model that builds upon the results of an agent‐based traffic simulation. All of these sub‐models have two important characteristics in common. First, they are built “bottom‐up,” which means that the central element of modeling are individual entities (e.g., individual buildings or individual households). Second, all sub‐models source their input data from Swiss national registers or other publicly accessible databases. This will help to keep the models transparent, up‐to‐date, and will result in comparable results for different regions by ensuring consistent modeling. Furthermore, similar databases are also maintained in other countries, making the generic modeling concepts of this article also interesting for other nations.

We will demonstrate the feasibility of the overall model by its application to Switzerland. More precisely, the system boundaries will comprise all private households consisting of the permanent population of Switzerland (this excludes, e.g., seasonal workers, persons in prisons, or residents of retirement homes). The functional unit for the life cycle assessment (LCA) will correspond to 1 year of household consumption and all computations refer to the year 2013 wherever possible. Note that we will only consider environmental impacts that can be traced back to the individual behaviors of households. This means, for instance, we will not redistribute government‐induced impacts to households. As a pivot database for the households we will use STATPOP 2013 (Statistics of the Population and Households of Switzerland) (Bundesamt für Statistik [BFS], 2014c). The STATPOP data provides socio‐demographic information about each household and each person in Switzerland, including gender, age, marital status, and the geographical coordinates of living. In total, 110 attributes for approximately 8 million persons and 62 attributes for 3.5 million private households are available (see also the Supporting Information).

### Building energy model

For assessing the residential energy needs of households, we chose to integrate the physically based building energy model of Buffat, Froemelt, Heeren, Raubal, and Hellweg (2017). This model estimates the demands for space heating and hot water of each residential building within a specified region. It builds upon the preliminary model of Saner et al. (2013), which itself was subjected to an in‐depth analysis by Froemelt and Hellweg (2017) and proved to be a promising approach for large‐scale building stock investigations. Buffat and colleagues improved this model by integrating large‐scale geographic information, particularly, by including 3D‐building geometries derived from laser‐scanning data. This also enabled the consideration of location‐specific shadowing, encompassing the effects from neighboring buildings and topography. The final building model establishes simplified energy balances for each residential building in Switzerland as a function of time, climate data, building characteristics, building statistics, surrounding topography, and 3D‐building geometries. One important reason for choosing this model is its basis on physical principles and its bottom‐up and component‐based structure. This renders the model capable of evaluating detailed refurbishment scenarios, for instance, by assessing the effects of physical measures such as the insulation of individual building components (e.g., walls, windows, or roofs) (Heeren, Jakob, Martius, Gross, & Wallbaum, 2013; Swan & Ugursal, 2009).

From the comprehensive Monte Carlo simulation, which is integrated in the building model, we retrieved the median estimate of space heating and hot water demand for each building. These model results were then assigned to individual households in a straightforward manner since the STATPOP data indicates in which building a household lives. In case of multiple dwellings in the same building, the building's energy demand was allocated based on the household's apartment area, which was extracted from the Swiss Building and Dwellings Statistics (BDS) (BFS, 2014b).

### Mobility model

Similar to the reasons for choosing a physically based building energy model, we assessed the mobility demand of households building upon the results of an agent‐based simulation. This link facilitates the implementation of different mobility scenarios for evaluating potential policy measures in future research. The agent‐based transport simulation framework MATSim (Multi‐Agent Transport Simulation; Horni, Nagel, & Axhausen, 2016) was applied to Switzerland to reproduce the mobility patterns of the Swiss population (Bauer et al., 2016). By providing spatially and temporally resolved information on chosen traffic modes and driven routes for each agent, this framework is well suited to model geographically disaggregated mobility demands. Since agents are statistical representatives of the population in a certain region, their mobility patterns were then assigned to actual STATPOP household members based on spatial information and several personal characteristics in a partially randomized optimization approach (see Supporting Information S1).

### Consumption‐archetypes model

Demand profiles of consumption archetypes from Froemelt, Dürrenmatt, and Hellweg (2018) were used to quantify the households' purchases in the areas of food, consumables, and other goods and services. We extensively exploited the Swiss Household Budget Survey (HBS) (BFS, 2013a) with machine learning and data mining techniques (Froemelt, Dürrenmatt, & Hellweg, 2018). The HBS delivers comprehensive information on the characteristics, socio‐economic conditions, expenditures, and bought quantities of the surveyed Swiss households (about 500 attributes for each household; see Supporting Information S1). The applied two‐stage clustering allowed for considering consumption data along with socio‐economic parameters and thus grouped households with similar living conditions and comparable behaviors. Thereby, 28 different consumption‐based archetypes representing typical household behavior patterns were recognized. The vectors of these archetypes do not only comprise estimates for expenditures, but also for purchased quantities, income, and possession of durable goods. In contrast to other studies (e.g., Bin & Dowlatabadi, 2005; Druckman & Jackson, 2008; Jones & Kammen, 2011; Lenzen et al., 2006; Weber & Matthews, 2008), this new approach does not rely on pre‐defined socio‐economic household segments, but derives its own household clusters, which are designed to take the observed variability of behavior within socio‐economic groups into account (Girod & De Haan, 2009). These clusters are thus well suited to study the nature and implications of different consumption behaviors in a coherent context of total household consumption. The study of Froemelt, Dürrenmatt, and Hellweg (2018) revealed that households in similar socio‐economic circumstances might differ in their consumption patterns and hence in both the total amount and composition of their environmental footprints.

### Interlinking the sub‐models to an overall model

The archetypes from the consumption sub‐model show a complete picture of demands for different population groups and are thus a predestined basis to interlink the three sub‐models (see Figure 1).

In spite of its richness in detail, the STATPOP dataset only focuses on socio‐demographic information and thus lacks important variables that were used for deriving the archetypes (Froemelt, Dürrenmatt, & Hellweg, 2018). Therefore, a direct allocation of archetypes to households by a classifier would not provide reliable matching or reasonable results. Due to this, we developed a new probabilistic classification approach which ensures the reproduction of a realistic variability of local household consumption. For this purpose, we employed a Random Forest Classifier (Breiman, 2001) based on the intersecting information of STATPOP (actual household) and HBS (archetypes), encompassing mainly geographic information and household characteristics. Furthermore, since the archetypes also provide estimates for residential energy and mobility demand, we integrated the already allocated results of the building energy sub‐model (Section 2.2) and the mobility sub‐model (Section 2.3) as additional household attributes into the training of the classifier. We decided to use a Random Forest Classifier in our study because of its generally good and robust performance (see also Supporting Information S1 and, e.g., Breiman, 2001; Fernández‐Delgado, Cernadas, Barro, & Amorim, 2014; Kuhn & Johnson, 2013). After calibration, the classifier computed the probabilities of belonging to a certain consumption archetype for each STATPOP household. Finally, one of the 28 archetypes was assigned to each STATPOP household in a random sampling process based on the household's individual probabilities. Since the computation of the probabilities also comprises the results of the two sub‐models for building energy and mobility demand, classifying a household as a certain archetype implicitly interlinks all three sub‐models and simultaneously preserves the context of total household consumption by maintaining the interrelations between consumption areas. The final overall model is highly resolved in space and detail by estimating individual consumption and income profiles with about 400 categories in both physical and monetary units for all 3.5 million Swiss households.

The technical details about the Random Forest Classifier are described in Supporting Information S1. In short, the tuning phase considered different performance metrics in an internal 10‐fold cross‐validation process on a 90% training set. The 10% left out sample was then used as a test set and to calibrate the probabilities (Kuhn & Johnson, 2013; Niculescu‐Mizil & Caruana, 2005; Pedregosa et al., 2012). Moreover, measures were deployed to prevent problems with class imbalance and therefore to account for the frequency of the archetypes' occurrence (e.g., stratified splitting in the cross‐validation procedure or prevalence weighting of archetypes within the classifiers (Kuhn & Johnson, 2013; Pedregosa et al., 2012).

### Environmental assessment

In the final modeling step, the environmental consequences induced by the quantified consumption profiles of the STATPOP households were assessed. Thereby, we mainly built upon the comprehensive hybrid LCA framework of Froemelt, Dürrenmatt, and Hellweg (2018), which they developed to assess the environmental impacts of the archetypes. This LCA modeling is called hybrid because it sources environmental background data from two methodologically different, but complementary approaches: environmentally extended input–output models (here: EXIOBASE (EXIOBASE Consortium, 2014; Wood et al., 2015)) and process‐based life cycle inventory databases (here: ecoinvent (Ecoinvent Centre, 2016; Wernet et al., 2016) and Agribalyse (Koch & Salou, 2015)). For the overall model, we extended the existing LCA modeling of the archetypes to also include the households' transport and residential energy estimates provided by the two respective sub‐models. Moreover, Froemelt et al.’s LCA framework was already adjusted as much as possible to Swiss conditions of consumption, but we now also tried to refine further the modeling to the circumstances of individual STATPOP households. For instance, we extracted the energy carriers used for space heating and hot water production of individual buildings from BDS (BFS, 2014b), constructed a process model for cars based on the car fleet composition (BFS, 2014d) of the household's respective canton, and chose a wastewater treatment plant activity from the ecoinvent database that matches the size of the plant in the household's municipality (Bundesamt für Umwelt, 2012) (see Supporting Information S1).

Note that the applied LCA is able to provide a highly detailed resolution by subdividing environmental footprints into more than 200 categories. In addition, it is not limited to carbon footprints, but could also provide estimates of other environmental indicators (Froemelt, Dürrenmatt, & Hellweg, 2018).

## RESULTS AND DISCUSSION

### Model evaluation

Prior to analyzing and using the model results, the validity and plausibility of the overall model and its sub‐models needs to be discussed. Since the goal of our modeling framework is to provide a realistic (but not necessarily an exact) picture of environmental footprint variability induced by individual households within a certain area, we take this as a reference point to appraise the model.

The building energy model was subjected to an in‐depth comparison with measured data in (Buffat et al., 2017; Froemelt & Hellweg, 2017) and was deemed to be well suited as a large‐scale building energy stock model. Likewise, the applications of MATSim to Switzerland have been successfully evaluated in (Bauer et al., 2016; Meister et al., 2010). However, the extrapolation of the MATSim agents to the STATPOP households in the context of the overall model (cf. Section 2.3) has not yet been investigated. In an effort to evaluate how well the computed person‐kilometers reproduce the real variability, we compared differently aggregated results with the Swiss Mobility Microcensus 2010 (Bundesamt für Statistik, & Bundesamt für Raumentwicklung, 2012) (see Supporting Information S1). By generally deviating by less than 20% (mostly even less than 10%), it can be concluded that the mobility sub‐model provides reasonable contributions to the overall model. The derivation of the consumption archetypes from the HBS, which is representative for Switzerland (BFS, 2013b), made use of different internal evaluation metrics in several steps (Froemelt, Dürrenmatt, & Hellweg, 2018). Finally, the archetypes approach could also bear up against the juxtaposition with independent national statistics in Froemelt, Dürrenmatt, and Hellweg (2018).

Even though each of the three sub‐models delivers plausible results, the question remains if the extrapolation of the archetypes to actual households and the interlinkage of the three models still provides a realistic picture of household consumption. This shall be demonstrated along four arguments: first, we used internal evaluation procedures for tuning the classifier (cf. Section 2.5 and Supporting Information S1). For instance, the applied cross‐validation separates a part of the training dataset as a validation set (in 10‐fold cross‐validation this is repeated 10 times) and additionally, the tuned classifier was evaluated with a 10% held out test sample in a final step (see Supporting Information S1 for the final metrics). Second, and as outlined in Section 2.5, several counteractions were taken to prevent problems with class imbalance. These measures were successful and yielded 0.87 for both Pearson and Spearman correlation coefficients for the archetypes’ prevalence in the HBS and the overall model. This indicates that the overall model exhibits a similar frequency distribution of archetypes as in the original HBS data. Apart from the above‐mentioned internal validation mechanisms, a household‐by‐household comparison is impossible since no external primary data in this resolution is available. But as the goal was to provide a realistic (but not necessarily a precise) variability of household behaviors, we circumvented this problem by comparing differently aggregated expenditures and revenues of the model with the original HBS in a third evaluation step to better understand the plausibility of the model's disaggregation. As could be expected based on the second argument above, this resulted in a good agreement of data (see Supporting Information S1). Fourth, overall national statistics for energy‐, water‐, and waste‐related data as well as an independent income‐related dataset could be satisfactorily reproduced (see Supporting Information S1).

**Figure 2:**
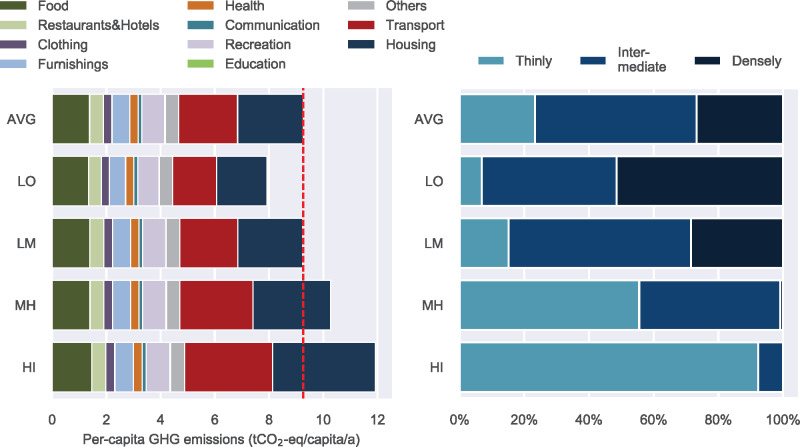
Results of the status quo analysis of municipal carbon footprints. (Left) Average carbon footprint of persons living in the four municipal clusters (LO: lowest impact municipalities [<10% percentile], LM: low‐to‐medium impact municipalities [10‐50%‐percentiles], MH: medium‐to‐high impact municipalities [50‐90%‐percentiles], HI: high impact municipalities [>90%‐percentiles]) as well as the Swiss average carbon footprint (AVG). The red dashed line is the Swiss average and shall support the comparison of the clusters with the AVG. (Right) Composition of the municipal clusters and the whole of Switzerland according to the DEGURBA classification of density (weighted by the number of persons living in the respective municipalities) (Eurostat, 2019). Underlying data used to create this figure can be found in Supporting Information S2. t, metric tons

As a final justification of the model results, we would like to relate the estimated life cycle GHG to previous studies: the model's consumption‐based total carbon footprint of Switzerland amounts to 9.3 tCO_2_‐eq/person/year (t, tonnes; see also Figure 2), while Girod and De Haan (2010) estimated 8.6 tCO_2_‐eq/person/year in a pure HBS‐based study and the top‐down assessment of Jungbluth, Nathani, Stucki, and Leuenberger (2011) found 11.0 tCO_2_‐eq/person/year. In all three approaches, housing, mobility, and food are identified as contributing the largest shares to the total footprints, which is also in line with many existing findings on an international level (e.g., Hertwich & Peters, 2009; Ivanova et al., 2016; Tukker & Jansen, 2006). The two Swiss studies above did have different underlying reference years, and we adjusted Jungbluth et al.’s original average of 12.8 tCO_2_‐eq/person/year for a better comparison with our approach, which underestimates impacts induced by health care, governmental activities, and educational services (as explained in Section 2.6 and in Froemelt, Dürrenmatt, & Hellweg, 2018; the consumption sub‐model only considers “*environmental impacts directly associated with a certain household's behavior*”). A possible explanation for the still lower average footprint of our bottom‐up model compared with Jungbluth's top‐down approach could be the use of consumer expenditure surveys (here: HBS), which might suffer from underreporting (Ivanova et al., 2017; Steen‐Olsen, Wood, & Hertwich, 2016). In line with this, the largest deviation can be found in the consumption area of food (2.1 vs. 1.4 tCO_2_‐eq/person/year). Furthermore, building construction is only partly covered in our approach since the HBS provides information on expenditures but not on investments (thus, although maintenance, certain refurbishments, and some building extensions are considered, the construction of a complete new edifice is not).

The internal evaluation measures and the attempts to assess the model's ability to reproduce the overall characteristics of national statistics reveal that the model points in the right direction and is able to deliver plausible and realistic assessments of individual households' consumption footprints.

### Status quo analysis

We will demonstrate some of the abilities of the overall model by means of a status quo analysis of municipal carbon footprints. In accordance with the functional unit defined in Section 2.6, the municipal carbon footprints are an aggregation of life cycle GHG induced by private households residing in a certain municipality and do not consider government activities. We chose a municipal viewpoint since we particularly would like to target local authorities with our model. Furthermore, we refrained from analyzing individual households since this is part of future research and would—in the current state—lead to similar results as in Froemelt, Dürrenmatt, and Hellweg (2018). Finally, note that the applied LCA enables for the assessment of all environmental indicators that are supported by the underlying databases (see Section 2.6). However, because of its prominence and for the ease of comparison with other studies, we will restrict ourselves to only presenting life cycle GHG and in the main consumption areas.

In a first step, we ranked all 2352 Swiss municipalities (Bundesamt für Landestopografie (swisstopo), 2014) according to their average per‐capita carbon footprint and then grouped the municipalities into four clusters taking the 10%, 50%, and 90% percentiles as borders (see Supporting Information S1): cluster LO comprises the 10% of the municipalities with the lowest emissions, low‐to‐medium emission municipalities belong to cluster LM (10–50% percentiles), cluster MH stands for medium‐to‐high communities (50–90% percentiles) and the 10% highest emitting municipalities are members of cluster HI. The average carbon footprints of people living in these different municipal clusters are presented in Figure 2 and compared to the Swiss average.

In a second step, we compared different statistics and characteristics of the grouped municipalities, including the estimated environmental impacts, the modeled incomes, household size and age structure, public transport services, per‐capita living area, and building stock statistics. In addition to a visual judgement of the distributions of these continuous variables, we applied one‐way ANOVA tests (Fisher, 1973) and used the resulting test statistics (*F*‐values) as quantitative indicators of how distinct the four municipal clusters are in the variable under consideration. Apart from these continuous variables, we also investigated categorical attributes such as the classification of municipalities according to DEGURBA (“Degree of Urbanisation”—definition of the European Union which classifies municipalities into “thinly populated area,” “intermediate density area,” and “densely populated area”; Eurostat, 2019), the classification into rural, urban cores, or agglomeration communities by BFS (2014a) as well as Swiss major regions (all statistics are presented in Supporting Information S1). Note that only income and environmental impacts are model results, while all other variables are retrieved from official statistics. As shown by many previous studies, income is a main driver for environmental footprints (e.g., Baiocchi et al., 2010; Ivanova et al., 2017; Jones & Kammen, 2011; Tukker et al., 2010; Weber & Matthews, 2008; Wiedenhofer, Smetschka, Akenji, Jalas, & Haberl, 2018). However, external income statistics is missing on a municipal level. Therefore, we laid a special focus on the evaluation of the model's income distribution (see Supporting Information S1) and included the modeled incomes in the analysis.

A thorough examination of above variables reveals that the four municipal clusters mostly differ in environmental consequences induced by housing, transport, and recreation, if only environmental impacts are considered. This could be expected to some extent since these three categories belong to the four most important consumption areas contributing to the total carbon footprint (see Figure 2). Even though food also appertains to these top emitting areas, it is less distinctive for forming the four municipal clusters. Being a basic need, the differences in food impacts among households are apparently less pronounced than for other consumption categories. However, the spread within the four clusters is large for all consumption areas. For instance, some municipalities with low per‐capita housing emissions still belong to HI.

Among the continuous variables, the following five findings are: (1) The average gross income per inhabitant is one of the most distinctive variables for the four municipal clusters. Thereby, higher per‐capita incomes tend to result in higher municipal carbon footprints. (2) Another essential factor, in which the four clusters differ, is the portion of buildings built before 1919 in a municipality. These old edifices show poorer insulation than new buildings by trend (Wallbaum, Heeren, Jakob, Martius, & Gross, 2010). In line with that, the percentage of new buildings appears to have a certain reductive effect on the municipal footprints. (3) The share of “families” (households with persons aged <25 and 25–64 years) in a municipality apparently also co‐determines substantially the municipal carbon footprint (the higher the share, the lower the municipal footprint). Moreover, the share of households with persons aged 65+ shows some importance, but with contrary impact on the municipal footprint. This corresponds well with the findings of Froemelt, Dürrenmatt, and Hellweg (2018), which showed that—except for very‐high‐income families—families show lowest per‐capita GHG compared to other archetypes; this is most probably due to effects of economy of scale and is similarly confirmed by Underwood and Zahran (2015) and Weber and Matthews (2008). (4) Related to the previous finding, the percentage of 2‐persons households compared to 3+‐persons households seems to be another important determinant for environmental impacts. For instance, HI as well as MH show clearly higher shares of 1‐ and 2‐persons households and lower shares of 4/4+ households, emphasizing again the effects originating from the household size. Note that a higher number of household members might not only result in efficiency gains, but can also lead to lower per‐capita incomes for larger households. Therefore, findings 1, 3, and 4 adopt different perspectives, but exhibit certain interrelations to each other. (5) Finally, the share of people in a municipality with poor public transport services increases the municipal carbon footprint.

Looking at the categorical classification of municipalities, two attributes are striking: the DEGURBA classification and the subdivision into rural, urban core, and agglomeration municipalities (see Figure 2 and Supporting Information S1). Almost all persons that live in areas classified as “densely populated” live in LO or LM municipalities, while HI mainly consists of persons living in “thinly populated” areas. This is in accordance with previous studies revealing that households in dense urban areas tend to have lower impacts, especially in the domains of mobility (shorter distances, better public transport services) and housing (smaller apartments) (e.g., Baiocchi et al., 2010; Jones & Kammen, 2014; Tukker et al., 2010; Wiedenhofer et al., 2018).

**Figure 3:**
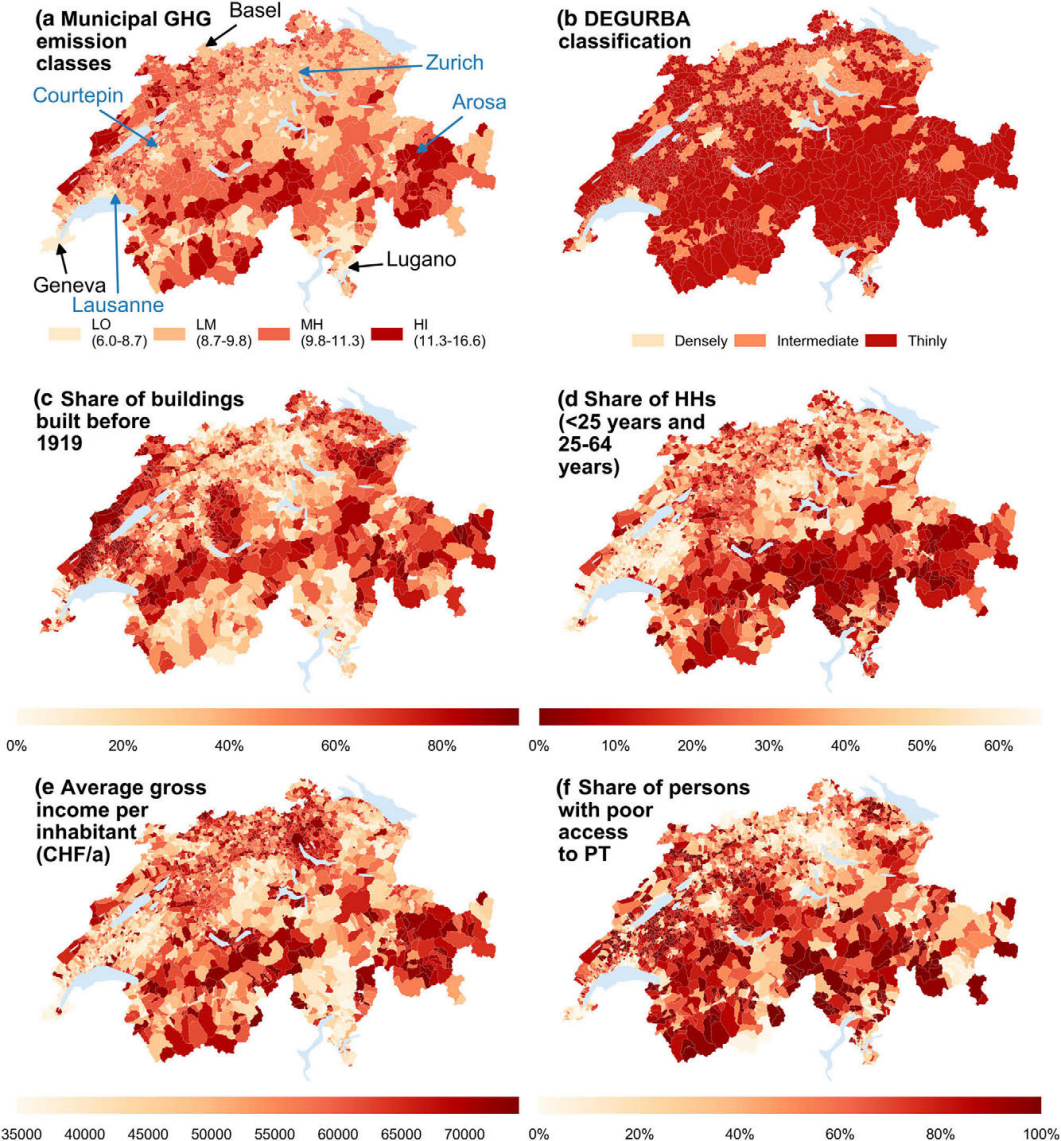
Maps of Switzerland (municipal boundaries provided by Bundesamt für Landestopografie (swisstopo), 2014. (a) Municipal clusters according to average per‐capita GHG emissions. Numbers in the legend refer to the cluster boundaries and are in tonnesCO_2_‐eq per capita and year. Municipalities labeled in blue are discussed in the text, while those labeled in black are meant for the reader's orientation. (b) DEGURBA classification of municipalities. (c) Share of buildings built before 1919 in % of all buildings in a municipality. (d) Share of households (HH) with persons aged <25 years and 25‐64 years in % of all households residing in a municipality. (e) Modeled average gross income per inhabitant. (f) Share of persons living in areas with poor access to public transportation (PT). “Poor access to PT” is defined as an area with poorest public transport services according to the classification of the Federal Office for Spatial Development (ARE [Bundesamt für Raumentwicklung, 2017]). Note that the color maps for b) and d) are inverted in order to better recognize correlations with (a), (c), (e) and (f). Underlying data used to create this figure can be found in Supporting Information S2

Figure 3 maps the municipal clusters and five of the most interesting above‐mentioned variables. A closer analysis of these maps, but also of the diagrams in Supporting Information S1, reveals that the variables considered sometimes correlate and sometimes counteract, leaving the municipal carbon footprint the result of an ensemble of interacting determinants. For instance, the municipality of Lausanne (fourth largest city of Switzerland, 128,070 inhabitants) is classified as an LO, which seems plausible in view of its DEGURBA classification of “densely populated” and its high share of people in areas of excellent public transport services. The latter two attributes are also true for Zurich (largest city of Switzerland, 368,351 inhabitants); however, Zurich belongs to LM, which is likely due to its very high share of 1‐ and 2‐persons households and its low share of families. Both municipalities exhibit relatively high incomes per inhabitant showcased by 94% (Lausanne) and 98% (Zurich) of all Swiss municipalities having lower per‐capita incomes. Despite the importance of per‐capita income, this alone is obviously not the only decisive factor.

Contrary to these two urban municipalities, Arosa (small Alpine touristic village, 3,030 inhabitants) is situated in a thinly populated area and has poor public transport connections along with high mobility emissions. It further has a rather low share of families, but a high share of aged households, which also corresponds to a comparably high share of 1‐ and 2‐persons households. In addition, the average gross income per inhabitant is almost equal to Lausanne (94% percentile rank). Becoming a member of the HI cluster is thus a logical consequence according to our above observations. Courtepin (small municipality in the Swiss midlands, 3,501 inhabitants) is also located in a thinly populated, rural area. Despite poor public transport services and comparably high transport emissions, it is labelled as LO. This is probably due to a very high share of families, a rather low income per inhabitant (8% percentile rank) and a very low portion of buildings built before 1919. Note that these were just four simplified cases to exemplify possible effects of the variables considered. We are aware that other factors might also influence the environmental impacts of a municipality and that deeper analyses are needed to identify causal relationships and possible drivers of footprints. In any case, the opposing effects of above variables emphasize the importance of aiming for a spatially highly resolved modeling framework that accounts for local circumstances.

**Figure 4:**
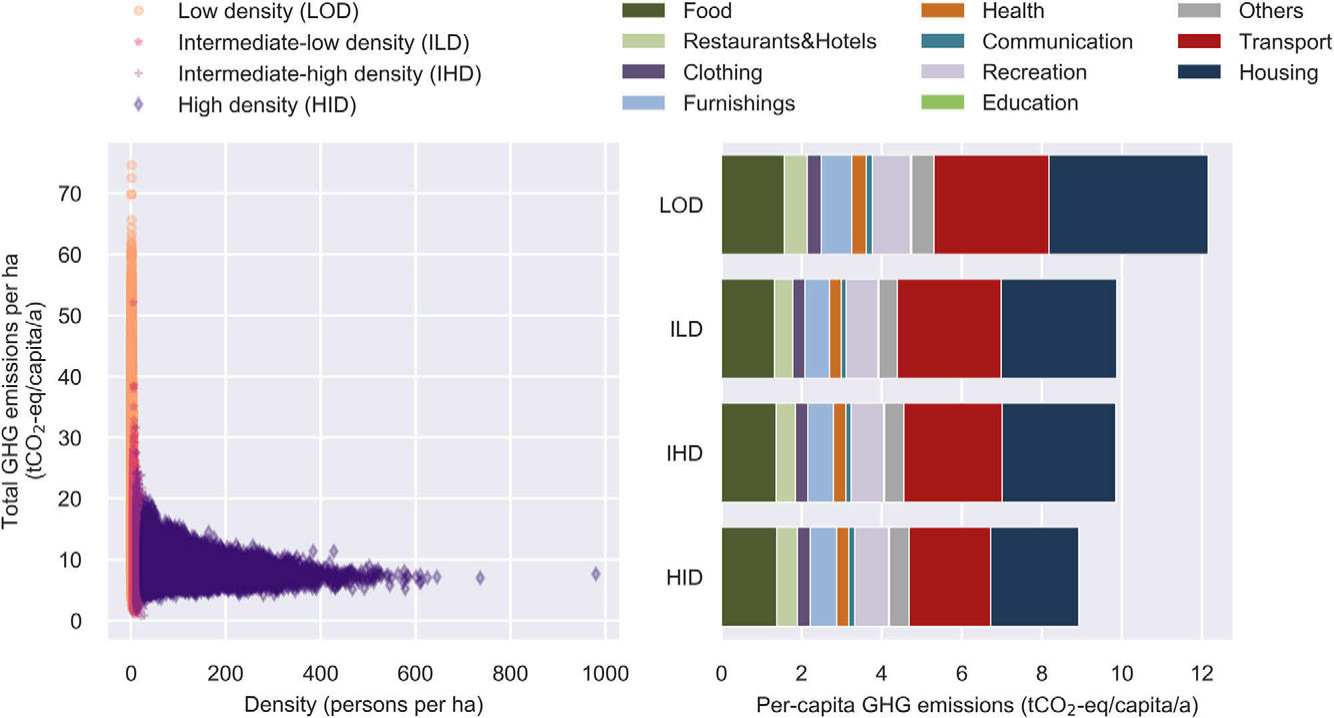
Results of the analysis of the relationship between population density and environmental impacts on a hectare‐basis. (Left) Relating the per‐capita carbon footprint of hectares to number of persons per hectare; (Right) Average per‐capita carbon footprint of people living in hectares of different densities (classification according to quartiles of persons per hectare). Underlying data used to create this figure can be found in Supporting Information S2. t, metric tons

Moreover, the findings above reveal that density (so far only taken into account as DEGURBA classification) is an important characteristic if municipal carbon footprints are considered. Therefore, we decided to further investigate the effect of density on environmental consequences. Profiting from the high spatial resolution of our model, we analyzed the relationship between persons per hectare (100 × 100 m) and per‐capita GHG (see Figure 4). Such a geographically highly resolved analysis is easily facilitated by adopting a household‐centered perspective in our model. In order to provide a better grasp of the environmental impacts caused by persons residing in different density classes, we used quartile groups of the number of persons per hectare (LOD: 25% of hectares with lowest density; ILD: low‐to‐intermediate density [25–50%]; IHD: intermediate‐to‐high density [50–75%]; HID: 25% of hectares with highest density of persons per hectare). Figure 4 shows a clear trend that increasing density is associated with decreasing environmental impacts. However, this effect obviously levels off at a certain density. Furthermore, Figure 4 reveals that some hectares show the lowest GHG along with comparably low density. This shows again that density is only one of many factors influencing environmental impacts, and it calls for further investigations in future research. Thereby, not only number of persons per hectares should be considered, but also other density indicators, such as access to various services. By examining the footprint compositions in Figure 4, one can observe that all four density groups exhibit similar amounts of emissions in the different consumption areas except for housing and mobility. As indicated above, this was not unexpected since both categories are inherently coupled with density to some degree (low density usually means longer transport distances, poor public transport services, and larger per‐capita living areas).

### Limitations of the model

Setting up a detailed modeling framework is an endeavor that inherently involves a multitude of limitations and uncertainties; most of them have already been discussed in the publications associated with the sub‐models. Here, we would like to mainly point to the assumptions used for interlinking the sub‐models and assigning archetypes to households. While it might be a strong but reasonable assumption to link the models by means of overlapping model results (here person‐kilometers and heating demand), we employed different countermeasures to attenuate negative effects of this presumption. These includes working with quantiles (see Supporting Information S1) or with probabilities rather than with absolute amounts and direct allocations. These same measures also bridge knowledge gaps between STATPOP and HBS, facilitating a realistic estimate for each household in a region. The above evaluation efforts show that our measures are taking hold and the modeling framework is able to provide reasonable results.

The chosen system boundaries can be regarded as a further limitation of the model, for example, the restriction to private households, the disregard of governmental consumption, and the consideration of only consumption activities that can be directly tracked to household behavior. The final computed environmental impacts might further be affected by the assumptions and simplifications that are needed to apply LCA.

Facing the above limitations and the fact that detailed external data for in‐depth model validations is missing, the development of an uncertainty analysis framework for our model is an important next step. Thereby, we would like to emphasize that for the building energy model, a comprehensive Monte Carlo simulation has already been implemented. The presented assignment of archetypes to households and the allocation of MATSim agents to household members can be regarded as one realization of a Monte Carlo simulation. A similar uncertainty framework as the one for the building sub‐model could be directly introduced, but would currently face computational challenges. In addition, uncertainties originating from the life cycle inventories and from the archetypes approach will be considered in future research.

In any case, if our framework will be used by local authorities for a particular municipality, the model can be easily adjusted and tuned with better local data.

## CONCLUSIONS

By the careful application of data mining and machine learning techniques and by interlinking various sub‐models—each of them originating from different modeling disciplines—we were able to generate highly resolved local consumption‐related data based on existing nationwide databases. Note that the employment of machine learning techniques is not limited to the current article. Also in our previous work for the sub‐models and specifically for deriving the consumption‐based archetypes as well as for handling the big GIS data in the building energy model, different tools from the machine learning toolbox have been applied, for instance, self‐organizing maps (Kohonen, 1990; Kohonen, 2001), various clustering algorithms (e.g., Lloyd, 1982; Ward, 1963), LASSO Regression (Tibshirani, 1996), and Random Forest Regression (Breiman, 2001). We hope that our efforts may ignite and encourage other researchers in the field of industrial ecology to consider the deployment of data mining tools.

The bottom‐up household‐centered character of the model allows for informing policymakers on different scales. This is of high importance since certain environmental measures cannot be directly implemented by municipalities, but require the involvement of national or cantonal authorities (Froemelt, Mauchle, et al., 2018).

The present article demonstrates the feasibility of the model through its application to Switzerland, shows the plausibility of the results, and exemplifies the usefulness of the model outcomes in the context of an investigation of the relationship between population density and environmental impacts as well as a status quo analysis of municipal carbon footprints. The latter revealed per‐capita gross income, population density, buildings' age, and household types as possible drivers of municipal carbon footprints. Although it is of high interest to investigate and understand these tendencies in the big picture, the aim and the abilities of the model platform go beyond these kinds of analyses. This is particularly important given that overall trends do not always apply to individual households nor to individual municipalities as was demonstrated with above case studies. Having tailored information about the variability of local consumption patterns at hand, household target groups can be identified and targeted measures designed. By applying insights of behavioral economics and psychology to the detailed household profiles of this study, this could also involve novel policy instruments such as personalized messages to successfully raise awareness and to guide households toward more sustainable consumption behaviors.

## OUTLOOK

Apart from the aforementioned development of an uncertainty framework, we aim to establish transdisciplinary collaborations with municipalities in order to test the model for its real‐world capability and to gain insights into the development of policy instruments based on the information provided by the model. The ability to evaluate policy scenarios is especially important in this regard. This aspect was thus considered in the model development and in the choice of the sub‐models. For example, the physical‐ and component‐based approach of the chosen building energy model will allow for computing detailed refurbishment scenarios, such as the effects of physical measures applied to specific buildings, specific components (e.g., roofs, walls, or windows), specific geographical regions, or specific homeowners (e.g., target groups which were identified due to their consumption behavior and living conditions). Likewise, the link to MATSim enables for the consideration of future mobility scenarios such as electric car penetration or autonomous vehicle systems (Frischknecht et al., 2018). In the context of assessing scenarios, we will also attempt to capture rebound effects of different policy applications. This could either be achieved by coupling our model with macro‐economic models or via further advancing the archetypes‐approach (e.g., by sub‐clustering and reassigning archetypes).

To complement the consumption perspective of our present model, the modeling of a production perspective should be envisaged in future work. The responsibility of households makes sense conceptually and they can definitely be considered as key actors for reducing environmental impacts. However, since households do not have full control of the supply chains serving them (O'Rourke & Lollo, 2015; Spangenberg & Lorek, 2002), an approach to assess environmental impacts of local industry and trade would facilitate local policymakers to also devise measures for industries located in their sphere of influence.

Although many improvement and future research efforts are possible, we would like to conclude that the current model can already provide a comprehensive basis to support policymakers in understanding locally occurring consumption behaviors. It also represents a virtual platform to evaluate policy scenarios intended to lower environmental impacts from household consumption.

## Supplementary Information

**Supporting Information S1**: This supporting information provides: 1) An overview of the open‐source software used; 2) A brief data description of HBS and STATPOP (main databases used); 3) Details on the mobility sub‐model; 4) Details on the interlinking of the different sub‐models, including technical details on the applied Random‐Forest‐Classifiers; 5) A description of the applied LCA‐modeling; 6) Results of the model evaluation; and 7) Further and more detailed results of the status quo analysis. (PDF 6.42 MB)

**Supporting Information S2**: This supporting information provides: 1) Details on the per‐capita carbon footprints and footprint compositions of all Swiss municipalities; 2) Data plotted in Figure 2 of the main text; 3) Data plotted in Figure 3 of the main text; 4) Data plotted in Figure 4 of the main text; 5) Data of the large tables presented in supporting information S1. (XLSX 9.03 MB)
