# Supplementary material for: Machine learning based modeling of households: A regionalized bottom‐up approach to investigate consumption‐induced environmental impacts
Source: J Ind Ecol. 2019 Nov 24;24(3):639–52. doi: 10.1111/jiec.12969 (PMC13061828; doi:10.1111/jiec.12969)
Supplement: Supplementary file 1 — Supporting Information S1: This supporting information provides: 1) An overview of the open‐source software used; 2) A brief data description of HBS and STATPOP (main databases used); 3) Details on the mobility sub‐model; 4) Details on the interlinking of the different sub‐models, including technical details on the applied Random‐Forest‐Classifiers; 5) A description of the applied LCA‐modeling; 6) Results of the model evaluation; and 7) Further and more detailed results of the status quo analysis. (PDF 6.42 MB) [file 44498_2020_2403015_MOESM1_ESM.pdf]

SUPPORTING INFORMATION FOR:

Froemelt, A., Buffat, R. & Hellweg, S. 2019. Machine Learning Based Modeling of Households: A Regionalized Bottom-Up Approach to Investigate Consumption-Induced Environmental Impacts. *Journal of Industrial Ecology.*

Summary

This supporting information provides: 1) An overview of the open-source software used; 2) A brief data description of HBS and STATPOP (main databases used); 3) Details on the mobility sub-model; 4) Details on the interlinking of the different sub-models, including technical details on the applied Random-Forest-Classifiers; 5) A description of the applied LCA-modeling; 6) Results of the model evaluation; and 7) Further and more detailed results of the status quo analysis.

Table of Contents

1 Open-Source Software S1-3

2 Brief Data Description S1-3

3 Mobility Model S1-4

3.1 Multi-Agent Transport Simulation (MATSim) S1-4

3.2 Allocation of MATSim-Agents to STATPOP-Household Members. S1-5

4 Interlinking the Sub-Models by a Random-Forest-Classifier S1-5

4.1 Overview S1-5

4.2 Data Preparation S1-6

4.3 Tuning the Random-Forest-Classifiers S1-7

4.4 Handling of Outliers S1-11

5 LCA-Modeling S1-11

5.1 LCA-Modeling for the Mobility Sub-Model S1-12

5.2 LCA-Modeling for the Building Energy Sub-Model S1-13

6 Model Evaluation with External National Statistics S1-15

6.1 Mobility Sub-Model in the Context of the Overall Model Compared to the Swiss Mobility Microcensus S1-15

6.2 Consumption Sub-Model in the Context of the Overall Model Compared to the Swiss Household Budget Survey S1-17

6.3 Comparison with Further National Statistics S1-25

6.4 Comparison with Further Income Statistics S1-26

7 Status Quo Analysis S1-27

References S1-35

1 Open-Source Software

All computations for this article were performed with the following open-source software:

PostgreSQL (The PostgreSQL Global Development Group, n.d.) 10

Python (Python Software Foundation, 2017) 3.6.6

Python packages:

- Brightway2 (Mutel, 2017) (2, 3)
- SciKit-learn (Pedregosa et al., 2012) 0.19.1
- NumPy (Van Der Walt, Colbert, & Varoquaux, 2011) 1.15.1
- SciPy (Jones, Oliphant, Peterson, & et al., n.d.) 1.1.0
- Pandas (McKinney, 2010) 0.23.4
- psycopg2 (Varrazzo, 2017) 2.7.3.1
- Matplotlib (Hunter, 2007) 2.2.2
- Seaborn (Waskom et al., 2017) 0.9.0

2 Brief Data Description

Even though many large datasets underlie the results of this article, only two of them are discussed in more detail to provide a better grasp of the overall model's abilities: the STATPOP-data (Statistics of the Population and Households of Switzerland) (Bundesamt für Statistik (BFS), 2014c) and the HBS-data (Swiss Household Budget Survey) (Bundesamt für Statistik (BFS), 2013b), respectively.

- STATPOP is a register survey and provides socio-demographic information for each person and each household registered in Switzerland. Socio-demographic data comprises age, gender, marital status, country/municipality of origin, nationality, and more. In numbers:
  - 110 attributes for 7970185 persons
  - 62 attributes for 3540641 households

  Note that the STATPOP-data is coupled with the Swiss Building and Dwelling Statistics (Bundesamt für Statistik (BFS), 2014b). This provides detailed up-to-date building-specific data about the buildings and the apartments in which the households live, including information about geographical coordinates of the building, construction year, construction period, building area, number of stories, number of apartments, area of each apartment, number of rooms of each apartment, heating and hot water system as well as corresponding energy carriers.
- Households participating in the HBS report on periodic expenses, possession of durable goods, income, household characteristics and, in particular, on daily expenditures and quantities of goods bought during one month. The HBS used in the study of Froemelt and colleagues (Froemelt, Dürrenmatt, & Hellweg, 2018) refers to the years 2009 to 2011 and comprises 9734 households. In numbers (for each household):
  - 20 different durable goods
  - 8 income attributes (plus 4 on aggregated levels)
  - 19 household variables
  - 6 attributes for each household member

- ○ 356 consumption categories (plus 175 on aggregated levels) according to the United Nation's COICOP-classification (United Nations Statistics Division, 2017)
- ○ 92 categories of purchased amounts of liters or kilograms (plus 14 on aggregated levels).

3 Mobility Model

3.1 Multi-Agent Transport Simulation (MATSim)

As briefly outlined in the main body of the article, MATSim is an agent-based traffic simulation framework. While a detailed description of MATSim can be found in (Horni, Nagel, & Axhausen, 2016) and the application of this framework to Switzerland is documented in (Hirschberg (ed.) et al., 2016), we would like to give the reader a brief overview of the characteristics of the MATSim-results.

In a first step, a synthetic population of agents with initial daily activity plans was created based on the Swiss Microcensus on land-based mobility behavior (Bundesamt für Statistik (BFS) & Bundesamt für Raumentwicklung (ARE), 2012). Furthermore, the framework is equipped with a detailed Swiss road network, locations of, e.g., work, schools, shopping, or leisure, as well as with public transport timetables and routes. MATSim then applies an iterative approach based on a coevolutionary algorithm (Horni et al., 2016). Within one simulation step, each agent attempts to pursue his/her plan of activities. Thereby, he/she can choose different traffic modes and different routes to travel from activity A to activity B. The simultaneous actions of all agents induce a traffic flow along the network links and may cause traffic jams. After each simulation step, the execution of the agent's plan is evaluated by a utility function. The agent gets, for instance, a higher score if he/she is able to perform all planned activities, if he/she gets to work on time or if travel times are reduced. For the next iteration step, the plans can be adjusted or a previously executed plan can be re-selected. After several iterations, the overall utility function (sum of the individual agents' utility functions) will finally converge. This does not mean that an optimal solution is found for all agents, but after this point, the overall system will not change significantly anymore.

MATSim-implementations for Switzerland were successfully validated (Meister et al., 2010).

MATSim covers the following land-based traffic modes (note that air travels and other forms of transport will be covered by the consumption sub-model): car, trolley bus, urban bus, long-distance train, high-speed train, regional train, metropolitan train, tram, bike and walking.

The simulated agents are characterized by age, gender and place of residence. Together with the plans from the Swiss Mobility Microcensus, they can be regarded as statistical representatives of the Swiss population; however, they do not directly correspond to actual STATPOP-persons. Therefore, as mentioned in the main body of the article and as described in section 3.2 below, we were required to allocate the agents to STATPOP-household members. This matching is of even higher importance since the MATSim-implementation of Switzerland only simulates a 10%-sample of the Swiss population due to the high computational burden of the MATSim-simulations. Therefore, the procedure of section 3.2 does not only allocate the agents' mobility demands to the households but simultaneously scales up the simulation from a 10%-sample to the full population.

It is important to note that MATSim only simulates one "typical" working day. However, since driven distances on weekends are comparable to working days, the annual mobility demand of households was estimated by multiplication of 365 days (Bundesamt für Statistik (BFS) &

Bundesamt für Raumentwicklung (ARE), 2012; Saner, Heeren, Jäggi, Waraich, & Hellweg, 2013).

3.2 Allocation of MATSim-Agents to STATPOP-Household Members.

The matching of agents to STATPOP-household members is performed based on the ideas of Saner and colleagues (2013). However, since the publication of (Saner et al., 2013), not only MATSim and its implementation for Switzerland has been improved, but we also re-developed and enhanced the matching-algorithm for the present study. In principle, the algorithm works with three mandatory constraints: the agent and the STATPOP-person needs to be in the same municipality (+500 m buffer zone), be of the same gender and be in the same age category. The age categories were chosen according to (Bundesamt für Statistik (BFS) & Bundesamt für Raumentwicklung (ARE), 2012). Within this pool of agents, the algorithm then allocates the agent with minimum difference in age and distance to the STATPOP-person. In case of ambiguity, the algorithm randomly samples among the closest agents.

The applied algorithm can be briefly explained by the following pseudo code:

```
For each Swiss municipality:
      Retrieve all agents within the municipal boundaries +500m buffer zone
      Retrieve all persons within the municipality
      For each person:
            Within a 100 m radius: look at all agents being of the same age
            category and the same gender of the person under consideration
            While the minimum age-difference is >5 years, increase the radius
            by 50m, but stop if radius > 500m
            From the found agents, take the ones with the lowest difference in
            age → if there are more than one agent: take the closest → if
            there are still more than one: randomly sample among the agents.
```

Please note that in Switzerland, some of the 2352 municipalities are very small and in remote Alpine areas. Therefore, the initial pool of agents retrieved for 29 municipalities were considered too small (<10 agents per gender category) to reproduce a reasonable mobility behavior. Limiting the model to only work with this initial pool of agents could lead to an overuse of some agents and also imply a risk of agents with overly high mobility demands ("outliers" / model artifacts) that would earn too much weight in these municipalities. Therefore, we adjusted the algorithm for those communities by continuously increasing the buffer zone. Since this could lead to a situation where an agent belonging to another municipality is always closest (by distance), we also introduced further constraints such as a minimum size of pool per person (not only per municipality), which might include relieving the age-category-constraints to some extent.

4 Interlinking the Sub-Models by a Random-Forest-Classifier

4.1 Overview

This section presents the technical details of the probabilistic assignment of consumption-based archetypes to STATPOP-households. The STATPOP-data (available for each household) provides socio-demographic data while the HBS-data (used to derive the archetypes) carries information on household characteristics and expenditure behavior. This means that the two datasets have only a certain overlap that renders the assignment of archetypes to

households fuzzy to some extent. As described in the main body of the article, we thus refrained from directly classifying households as a specific archetype. Instead, we trained Random-Forest-Classifiers (Breiman, 2001) with the HBS-data and then applied them to the STATPOP-data to compute the probabilities of a STATPOP-household to be member of a certain consumption cluster. The goal of the model was to provide a realistic – but not necessarily an accurate – estimate of the consumption-induced environmental footprints of individual households and a realistic – but not necessarily an accurate – picture of variability in impacts within a certain area. To achieve this goal, we randomly assigned an archetype to a STATPOP-household based on the probabilities computed by the classifiers. Since the consumption model provides intersecting estimates on mobility demand and housing demand with the other two sub-models, we were able to integrate this information in the computation of the probabilities. This implies that the assignment of archetypes implicitly interlinks all three sub-models and simultaneously preserves the context of total household consumption.

As will be outlined below, we actually trained two different classifiers; the first one for the case of complete information for a STATPOP-household and the second one to account for the case when sub-model results were missing (e.g. the household lives in a very new building for which the heating demand could not be computed by the building energy model due to unavailable data).

4.2 Data Preparation

The classifiers were trained based on the intersection of data between STATPOP (Bundesamt für Statistik (BFS), 2014c) and HBS (Bundesamt für Statistik (BFS), 2013b), which comprised information on the size of households, age of household members, gender of household members, marital status of household members and geographic location (altogether 33 attributes).

To couple the overall model with the mobility and the building sub-models, we further included the results of driven kilometers by car and the heating and hot water demand in MJ per year (see (Froemelt et al., 2018) for the estimate of heating and hot water demand for the archetypes as well as for the conversion of bought liters of diesel and petrol to driven kilometers). However, to get a more robust correspondence between the consumption archetypes and the two other sub-models, we decided to work with quantiles instead of absolute values. This procedure shall prevent problems with seasonality in the HBS-data and with potential outliers in general. While for the target data, the conversion to quantiles was straightforward, we decided to apply a similar procedure to HBS-data as was used by (Froemelt et al., 2018) to correct for seasonality in the HBS-data (note that the HBS-households are surveyed in different months which can imply seasonal effects). The procedure for the seasonality correction is explained as follows by (Froemelt et al., 2018): *"The dataset is partitioned into monthly subsets (e.g. "expenditures on fruits in January", "expenditures on fruits in February", etc.). In a next step, the original values are then replaced by its monthly quantile ranks. This means, if e.g. a household buys fruits for 7 Swiss Francs in January, this corresponds to a quantile rank of 0.42 in this month; or in other words: 42% of the expenditures for fruits of all households in January were below 7 Swiss Francs. Finally, the original value of 7 Swiss Francs is replaced by 0.42. Please note that this is a made-up example."* [and please note that for the present study, we did this procedure for driven kilometers and residential energy only and not for fruits and other consumption areas].

The STATPOP-data is complete for the considered households (note that we exclude, e.g., seasonal workers, persons in prisons, or long-term residents in hospitals or in homes for the elderly) and the mobility model allocates agents to these households by design. However, the building energy model does not provide estimates for all households in consideration. This is

due to several reasons. For instance, houses that were built in the year 2013 might be occupied by a household at the end of 2013, but might be missing in the underlying databases of the building energy model. In order to catch these cases, we needed to tune and calibrate two different classifiers: one that assumes a complete dataset and one which can be used in the case of housing data gaps and which is thus trained without housing-related data.

4.3 Tuning the Random-Forest-Classifiers

We decided to use a Random-Forest-Classifier because it is able to take non-linearities into account, is robust with regard to noise, but also with regard to non-informative features, is not overly sensitive to the tuned hyperparameters, does not require a laborious data pre-processing and shows generally a good performance (Breiman, 2001; Breiman & Cutler, 2003; Fernández-Delgado, Cernadas, Barro, & Amorim, 2014; Genuer, Poggi, & Tuleau, 2008; Kuhn & Johnson, 2013; Liaw & Wiener, 2002; Pedregosa et al., 2012). The hyperparameters of the Random-Forest (number of trees, maximum number of features considered in a split step, and quality criterion of a split) were tuned in a 10-fold cross-validation (Kuhn & Johnson, 2013) and based on the recommendations of (Breiman & Cutler, 2003; Genuer et al., 2008; Liaw & Wiener, 2002). Before the training started, a 10%-sample was separated with a stratified splitter which preserves the clusters' prevalence (Kuhn & Johnson, 2013; Pedregosa et al., 2012). This 10%-sample constitutes an important test-set for evaluation purposes, but is also needed for calibrating probabilities later on (Niculescu-Mizil & Caruana, 2005). The training on the 90%-original dataset was performed in all combinations of the following discrete states of the tuning parameters:

- Split quality criterion: "Gini-impurity" and "Entropy" (information gain)
- Number of trees: 1000, 2000, 3000
- Maximum number of features considered in a split: square root of number of features, twice the square root of number of features, half the square root of number of features, maximum number of features

To prevent problems with class imbalance, we followed the suggestions of (Kuhn & Johnson, 2013) and applied a stratified splitting also during the cross-validation process and simultaneously used the Random-Forest-inherent possibility to weigh the clusters based on their prevalence.

For evaluating the tuning process, we relied on different performance metrics: "out-of-bag"-error, accuracy, Cohen's Kappa, recall, precision, and f-score (Kuhn & Johnson, 2013; Pedregosa et al., 2012). Even though these metrics provide insights into the performance of a classifier from different perspectives, they did not contradict each other for evaluating the combination of tuning parameters in our case. Note that we weighted the performance metrics by the clusters' prevalence to give rare clusters more weight and to avoid class imbalance problems in the later use of the classifiers.

In order to select an appropriate combination of hyperparameters we followed a multi-step approach:

1. We extracted all above-mentioned metrics for both: the cross-validation step but also with the 10%-test-set.
2. Following the “one-standard-deviation”-rule (Kuhn & Johnson, 2013), all trained classifiers which had a performance value within one standard deviation from the model with the best performance were taken into consideration.
3. Among these subset of parameter-combinations, we then performed pair-wise comparisons between the simplest Random-Forest based on the "Gini-impurity" versus the

simplest Random-Forest based on the "Entropy"-criterion. The winner of the comparison was then juxtaposed to the next complex Random-Forest-model of the competing quality criterion.

4. Step 3 was repeated until one of the combinations outperformed all Random-Forest-models of the other split quality criterion.

➔ Selected model for no missing features:
    - Split quality criterion: "Gini-impurity"
    - Number of trees: 2000
    - Number of features to be considered in a split: twice the square root of number of features

➔ Selected model for missing features:
    - Split quality criterion: "Gini-impurity"
    - Number of trees: 1000
    - Number of features to be considered in a split: maximum number of features

The final performance metrics of these models are presented in Table S1-1.

Table S1-1: Final performance metrics for the two trained Random-Forest-Classifiers. "RF for no gaps" refers to the Random-Forest which is meant for STATPOP-households that have no missing values, while "RF for missing data" stands for the classifier which was trained for STATPOP-households for which no building-energy-results were available. [RF = Random-Forest, OOB = "Out-of-bag"-error]

	RF for no gaps		RF for missing data	
	Cross-validation	Test-set	Cross-validation	Test-set
**Cohen's Kappa**	0.63	0.63	0.60	0.58
**Accuracy**	0.65	0.64	0.62	0.60
**Precision**	0.63	0.63	0.61	0.59
**Recall**	0.65	0.64	0.62	0.60
**F-score**	0.64	0.63	0.61	0.59
**OOB**	0.65	0.65	0.61	0.61

In a post-processing step, the probabilities provided by these classifiers needed to be calibrated with the previously separated 10%-left-out-sample (Niculescu-Mizil & Caruana, 2005; Pedregosa et al., 2012). This is especially important for Random-Forest-Classifiers since they tend to underestimate probabilities close to 0 and 1 (Niculescu-Mizil & Caruana, 2005). The calibration was performed by Platt's sigmoid model (Niculescu-Mizil & Caruana, 2005). The two calibrated classifiers were then evaluated by a confusion matrix and a probability-heatmap respectively (see Figure S1-1 to Figure S1-4). Just as the tuning parameters above, also these evaluations showed satisfying results.

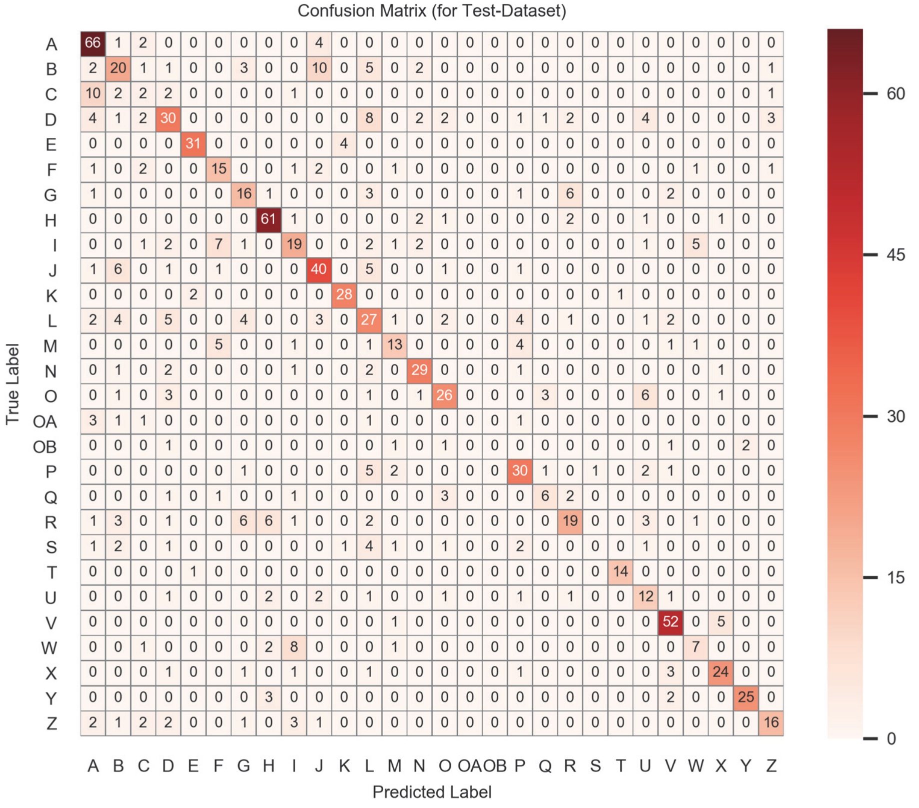

Figure S1-1: **Confusion matrix for the calibrated Random-Forest-classifier which is trained for households without missing data. Labels refer to the archetype-names of** (Froemelt et al., 2018).

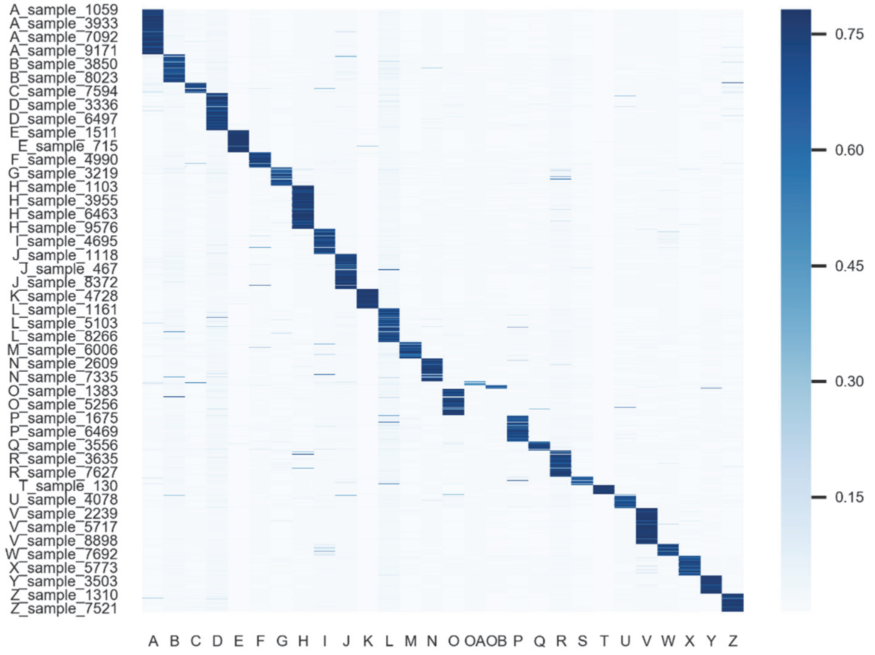

Figure S1-2: **Probability-heatmap for the calibrated Random-Forest-classifier that is trained for households without missing data. The heatmap illustrates the probabilities with which a training-sample household is classified as a certain cluster (reading example: column A shows the probabilities of being classified as cluster A. E.g., the probability for sample 1059, whose true cluster membership is A, is in the range of 0.75). Please note that we show all probabilities for all samples, but for keeping the matrix legible, we only label some indicative samples on the left-hand side.**

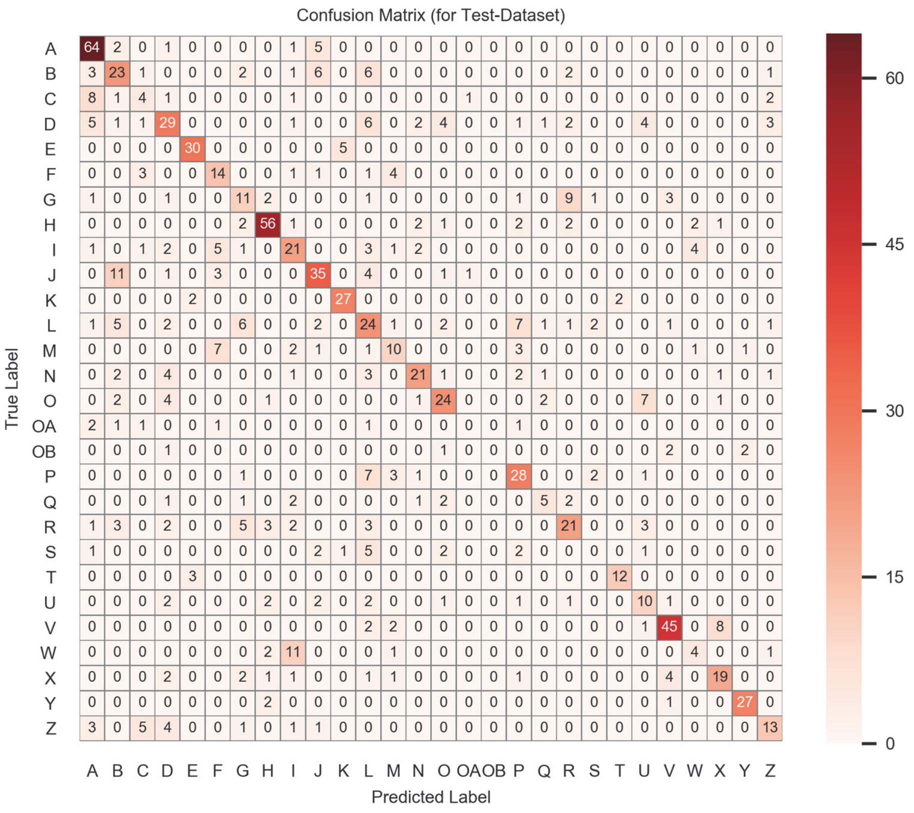

Figure S1-3: **Confusion matrix for the calibrated Random-Forest-classifier trained for households with missing building energy data. Labels refer to the archetype-names of** (Froemelt et al., 2018).

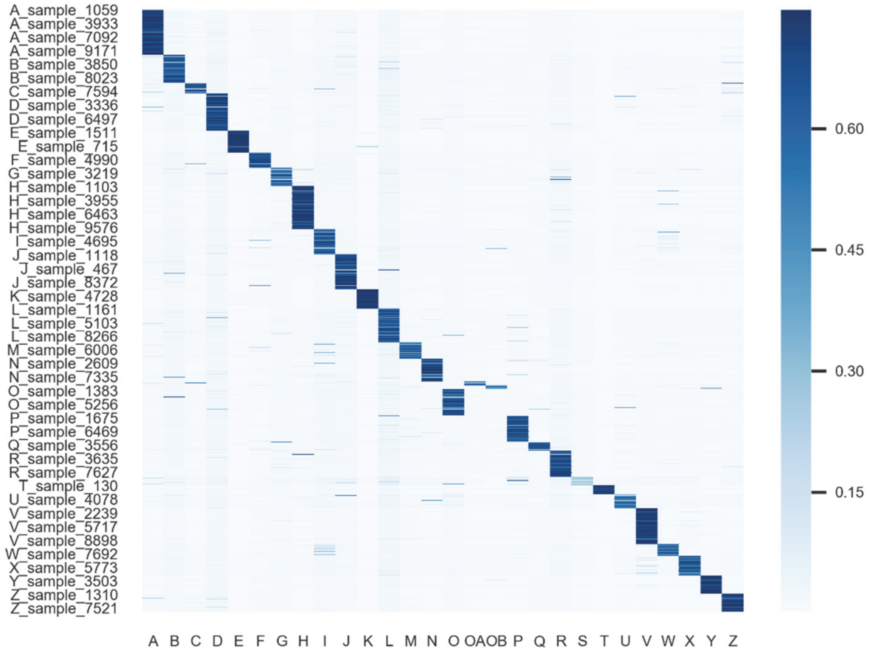

Figure S1-4: **Probability-heatmap for the calibrated Random-Forest-classifier trained for households with missing building energy data. The heatmap illustrates the probabilities with which a training-sample household is classified as a certain cluster (reading example: column A shows the probabilities of being classified as cluster A. E.g., the probability for sample 1059, whose true cluster membership is A, is in the range of 0.65). Please note that we show all probabilities for all samples, but for keeping the matrix legible, we only label some indicative samples on the left-hand side.**

4.4 Handling of Outliers

Preliminary results of the interlinked overall model revealed that outliers are present coming from the building energy sub-model or from the mobility sub-model. In the case of the building energy model, households with extraordinarily high energy demand could be traced back to apartments located in industrial zones. For instance, janitors sometimes live in an apartment that is integrated in a big industrial building. Depending on the data provided by the Federal Register of Buildings and Dwellings (FRBD) (Bundesamt für Statistik (BFS), 2013a), the building energy model then computes the heating demand for the whole building and allocates a much too high estimate to the janitor-family (note that this is just one example). In principle, the FRBD could indicate a mixed use of the building, but the respective attribute is often missing. Since it is thus difficult to set conditions to exclude such misestimates, we defined an upper threshold at which we replaced the model results by the residential energy estimate of the consumption model. This threshold was set as the average of the largest 50 heating energy demands provided by the HBS (Bundesamt für Statistik (BFS), 2013b). Please note that:

- 50 households is the minimum number of households with non-zero entries which can be regarded to be representative according to the Federal Statistical Office (Bundesamt für Statistik (BFS), 2013b).
- The heating energy demands were not directly available from the HBS, but were derived from expenditures and modeled in (Froemelt et al., 2018).
- We followed the instructions by the Federal Statistical Office and computed the average by weighting the households according to the representativeness-weights given by the Federal Statistical Office (Bundesamt für Statistik (BFS), 2013b).

With regard to the mobility sub-model, very few households were assigned an unrealistically high mobility demand. This was especially the case in remote areas where the pool of agents was – despite the measures taken in section 3.2 – limited and within these agents, some had extraordinary high demands, which can be considered simulation artifacts. These high-demand agents become a problem in particular if the same agent is attributed several times to the same household. Therefore, we decided to proceed similarly as for the building energy model above and defined an upper threshold in the same way.

Please note that replacing the building energy demands via the upper threshold only concerns 1.2% of all households, and the mobility sub-model results were replaced for 1.9% of all households. Furthermore, and as mentioned earlier, the building energy model did not provide estimates for all STATPOP-households (cf. section 4.2). These missing results were also imputed by the estimates of the consumption sub-model and concerns 9.5% of households.

5 LCA-Modeling

As explained in the main body of the article, we build upon the hybrid life cycle assessment (LCA) framework developed by (Froemelt et al., 2018) which sources environmental background data from ecoinvent v3.3 (Ecoinvent Centre, 2016; Wernet et al., 2016), Agribalyse v1.2 (Koch & Salou, 2015), and EXIOBASE v2.2 (EXIOBASE Consortium, 2014; Tukker et al., 2013; Wood et al., 2015). The Supporting Information of the article of (Froemelt et al., 2018) provides an in-depth explanation and discloses the whole LCA-modeling in an accompanying EXCEL-file. In the following, we will thus only focus on the extensions and changes made to the existing LCA-modeling.

5.1 LCA-Modeling for the Mobility Sub-Model

The results from the mobility sub-model for public transport demand could be directly coupled with different ecoinvent-activities (see Table S1-2). For cars, ecoinvent v3.3 provides several different activities. However, MATSim does not simulate a car fleet. This means, while we have detailed environmental data available for different cars, we do not know the car types used by the MATSim-agents. In order to create a process model for a car that takes the circumstances of a STATPOP-household into account as far as possible, we relied on generating cantonal car fleet mixes. For this purpose, we used the data from (Bundesamt für Statistik (BFS), 2014d) about the statistical car fleet compositions of each canton in Switzerland. The statistics are subdivided into car size (engine displacement), EURO-emissions code, and fuel. We then used these statistics to weigh the car-activities of ecoinvent (see Table S1-3) accordingly and generated "cantonal cars". Note that we allocated undefined EURO-code, EURO I, and EURO II to EURO III-car types, while EURO VI-cars were assigned to EURO V.

Table S1-2: LCA-modeling of public transport modes in MATSim.

MATSim-name	ecoinvent-activity	ecoinvent-location
Trolleybus/Low floor bus	transport, trolleybus	CH
Double-articulated bus	transport, regular bus	CH
Mid bus	transport, regular bus	CH
Bus	transport, regular bus	CH
Night train	transport, passenger train, long-distance	CH
Metro Lausanne	transport, passenger train, urban	CH
Regional train	transport, passenger train, regional	CH
City railway	transport, passenger train, urban	CH
Min bus	transport, regular bus	CH
Min tram	transport, tram	CH
Max LD train	transport, passenger train, long-distance	CH
Mid LD train	transport, passenger train, long-distance	CH
Min LD train	transport, passenger train, long-distance	CH
Max tram	transport, tram	CH
TGV 1-part	transport, high speed train	FR
Small LD train	transport, passenger train, long-distance	CH

In order to convert vehicle-kilometers (functional unit of ecoinvent for cars) to person-kilometers, we assumed an average load of 1.6 persons per car (Bundesamt für Statistik (BFS) & Bundesamt für Raumentwicklung (ARE), 2012).

It also needs mentioning that ecoinvent only provides a "global electric car". We took this process model and replaced the global electricity mix with the Swiss electricity mix.

Table S1-3: **Ecoinvent-activities** (Ecoinvent Centre, 2016; Wernet et al., 2016) **used for modeling the cantonal car fleet mixes. Note that ecoinvent's global electric car was adjusted by replacing the global electricity mix by the Swiss electricity mix.**

MATSim-name	ecoinvent-activity	ecoinvent-location
car	transport, passenger car, small size, petrol, EURO 3	RER
car	transport, passenger car, small size, petrol, EURO 4	RER
car	transport, passenger car, small size, petrol, EURO 5	RER
car	transport, passenger car, medium size, petrol, EURO 3	RER
car	transport, passenger car, medium size, petrol, EURO 4	RER
car	transport, passenger car, medium size, petrol, EURO 5	RER
car	transport, passenger car, large size, petrol, EURO 3	RER
car	transport, passenger car, large size, petrol, EURO 4	RER
car	transport, passenger car, large size, petrol, EURO 5	RER
car	transport, passenger car, small size, natural gas, EURO 3	RER
car	transport, passenger car, small size, natural gas, EURO 4	RER
car	transport, passenger car, small size, natural gas, EURO 5	RER
car	transport, passenger car, medium size, natural gas, EURO 3	RER
car	transport, passenger car, medium size, natural gas, EURO 4	RER
car	transport, passenger car, medium size, natural gas, EURO 5	RER
car	transport, passenger car, large size, natural gas, EURO 3	RER
car	transport, passenger car, large size, natural gas, EURO 4	RER
car	transport, passenger car, large size, natural gas, EURO 5	RER
car	transport, passenger car, small size, diesel, EURO 3	RER
car	transport, passenger car, small size, diesel, EURO 4	RER
car	transport, passenger car, small size, diesel, EURO 5	RER
car	transport, passenger car, medium size, diesel, EURO 3	RER
car	transport, passenger car, medium size, diesel, EURO 4	RER
car	transport, passenger car, medium size, diesel, EURO 5	RER
car	transport, passenger car, large size, diesel, EURO 3	RER
car	transport, passenger car, large size, diesel, EURO 4	RER
car	transport, passenger car, large size, diesel, EURO 5	RER
car	transport, passenger car, electric CH	CH

5.2 LCA-Modeling for the Building Energy Sub-Model

Since the Swiss Building and Dwellings Statistics (BDS) (Bundesamt für Statistik (BFS), 2014b) indicates the energy carriers used for a building, LCA-modeling can be done in a straightforward manner. The process models for the different BDS-energy-carrier-categories are listed in Table S1-4. The weights indicated in Table S1-4 correspond to the shares of the energy carrier technologies according to the final energy demands statistics of Switzerland (Bundesamt für Energie (BFE), 2015). In case of different technologies for the same energy carrier, either statistics from (Ecoinvent Centre, 2013) were used or equal shares were assumed. In the latter case, rough sensitivity analyses were performed and showed only insignificant changes in the LCA results. The BDS-energy-categories "no energy carrier/other energy carrier", "coal", and "district heating" were modeled by the average Swiss energy mix (Bundesamt für Energie (BFE), 2015), because appropriate ecoinvent-activities were missing. However, only 5.7% of all Swiss buildings fall into one of these categories.

Table S1-4: Process models for the LCA of housing energy with ecoinvent-activities (Ecoinvent Centre, 2016; Wernet et al., 2016)**. [BDS = Building and Dwelling Statistics** (Bundesamt für Statistik (BFS), 2014b)**]**

BDS-code	BDS-name (translated)	ecoinvent-activity	Weight
7200	no energy carrier	heat production, natural gas, at boiler condensing modulating <100kW' (megajoule, CH, None)	0.30
		'heat production, light fuel oil, at boiler 10kW, non-modulating' (megajoule, CH, None)	0.29
		'heat production, light fuel oil, at boiler 10kW condensing, non-modulating' (megajoule, CH, None)	0.29
		'heat production, mixed logs, at wood heater 6kW' (megajoule, CH, None)	0.03
		'heat production, mixed logs, at furnace 30kW' (megajoule, CH, None)	0.03
		'heat production, wood pellet, at furnace 9kW' (megajoule, CH, None)	0.03
		'heat production, softwood chips from forest, at furnace 50kW' (megajoule, CH, None)	0.02
		'heat production, hardwood chips from forest, at furnace 50kW' (megajoule, CH, None)	0.01
7201	fuel oil	'heat production, light fuel oil, at boiler 10kW, non-modulating' (megajoule, CH, None)	0.50
		'heat production, light fuel oil, at boiler 10kW condensing, non-modulating' (megajoule, CH, None)	0.50
7202	coal	heat production, natural gas, at boiler condensing modulating <100kW' (megajoule, CH, None)	0.30
		'heat production, light fuel oil, at boiler 10kW, non-modulating' (megajoule, CH, None)	0.29
		'heat production, light fuel oil, at boiler 10kW condensing, non-modulating' (megajoule, CH, None)	0.29
		'heat production, mixed logs, at wood heater 6kW' (megajoule, CH, None)	0.03
		'heat production, mixed logs, at furnace 30kW' (megajoule, CH, None)	0.03
		'heat production, wood pellet, at furnace 9kW' (megajoule, CH, None)	0.03
		'heat production, softwood chips from forest, at furnace 50kW' (megajoule, CH, None)	0.02
		'heat production, hardwood chips from forest, at furnace 50kW' (megajoule, CH, None)	0.01
7203	natural gas	heat production, natural gas, at boiler condensing modulating <100kW' (megajoule, CH, None)	1.00
7204	electricity	'market for electricity, low voltage' (kilowatt hour, CH, None)	1.00
7205	wood	'heat production, mixed logs, at wood heater 6kW' (megajoule, CH, None)	0.25
		'heat production, mixed logs, at furnace 30kW' (megajoule, CH, None)	0.25
		'heat production, wood pellet, at furnace 9kW' (megajoule, CH, None)	0.25
		'heat production, softwood chips from forest, at furnace 50kW' (megajoule, CH, None)	0.18
		'heat production, hardwood chips from forest, at furnace 50kW' (megajoule, CH, None)	0.07
7206	heat pump	heat production, borehole heat exchanger, brine-water heat pump 10kW' (megajoule, CH, None)	0.50
		'heat production, air-water heat pump 10kW' (megajoule, CH, None)	0.50
7208	district heating	heat production, natural gas, at boiler condensing modulating <100kW' (megajoule, CH, None)	0.30
		'heat production, light fuel oil, at boiler 10kW, non-modulating' (megajoule, CH, None)	0.29
		'heat production, light fuel oil, at boiler 10kW condensing, non-modulating' (megajoule, CH, None)	0.29
		'heat production, mixed logs, at wood heater 6kW' (megajoule, CH, None)	0.03
		'heat production, mixed logs, at furnace 30kW' (megajoule, CH, None)	0.03
		'heat production, wood pellet, at furnace 9kW' (megajoule, CH, None)	0.03
		'heat production, softwood chips from forest, at furnace 50kW' (megajoule, CH, None)	0.02
		'heat production, hardwood chips from forest, at furnace 50kW' (megajoule, CH, None)	0.01
7209	other energy carrier	heat production, natural gas, at boiler condensing modulating <100kW' (megajoule, CH, None)	0.30
		'heat production, light fuel oil, at boiler 10kW, non-modulating' (megajoule, CH, None)	0.29
		'heat production, light fuel oil, at boiler 10kW condensing, non-modulating' (megajoule, CH, None)	0.29
		'heat production, mixed logs, at wood heater 6kW' (megajoule, CH, None)	0.03
		'heat production, mixed logs, at furnace 30kW' (megajoule, CH, None)	0.03
		'heat production, wood pellet, at furnace 9kW' (megajoule, CH, None)	0.03
		'heat production, softwood chips from forest, at furnace 50kW' (megajoule, CH, None)	0.02
		'heat production, hardwood chips from forest, at furnace 50kW' (megajoule, CH, None)	0.01

Note that for the replacement estimates described in section 4.4 we also took the modeling of Table S1-4 into account and did not apply a Swiss energy mix as in (Froemelt et al., 2018).

Furthermore, for secondary homes, we applied the same modeling as in (Froemelt et al., 2018). For these apartments, no detailed information was available and we thus relied on a Swiss energy mix (see (Froemelt et al., 2018)).

Even though not directly related to the building energy model, we would like to point out that we also tailored wastewater treatment plant activities to the respective municipalities. From (Bundesamt für Umwelt (BAFU), 2012), we knew the size of the wastewater treatment plants in the different municipalities and were thus able to choose the correct size from the ecoinvent-database (Ecoinvent Centre, 2016; Wernet et al., 2016).

6 Model Evaluation with External National Statistics

As mentioned in the main body of the article, all sub-models have been tested and evaluated before they were integrated into the overall framework. However, special attention needed to be paid to the extrapolation of the agents from the mobility sub-model as well as to the assignment of archetypes to all actual households of Switzerland. This section of the SI aims thus at giving an impression of the plausibility of the model results by presenting a selection of comparisons of external national statistics with the results of the overall model. Note that in the main body of the paper, we additionally consider internal evaluation measures (see also section 4) and the frequency distribution of archetypes to evaluate the model results' reasonability.

6.1 Mobility Sub-Model in the Context of the Overall Model Compared to the Swiss Mobility Microcensus

In Table S1-5 the mean daily distance (kilometers per person per day) according to the mobility sub-model (see section 3) is compared with the Swiss Mobility and Transport Microcensus 2010 (Bundesamt für Statistik (BFS) & Bundesamt für Raumentwicklung (ARE), 2012) in different aggregations. Table S1-6 shows a similar comparison, but with regard to modal splits. Note that the cities of Geneva, Basel and Lugano were left out of the comparison due to their proximity to the national borders. The used MATSim-simulations do not consider routine cross-border trips (Hirschberg (ed.) et al., 2016). Therefore, the comparisons of model results with these cities' statistics were assumed not to provide reasonable results. Furthermore, we would like to point out that the modal splits are based on kilometers driven and that they relate car trips and public transportation trips to each other, but neglect bike and walk trips. Regardless, the latter two traffic modes are of less importance since they induce almost no greenhouse gas emissions.

The “PT-classes” in Table S1-5 and Table S1-6 are defined as follows: The Federal Office for Spatial Development subdivides the whole area of Switzerland into five classes according to the goodness of public transport (PT) access in a certain area. While PT-class A indicates access to excellent public transport services, a not-classified area means no or very infrequent public transport service (ARE (Bundesamt für Raumentwicklung), 2017).

Table S1-5: **Comparison of the mean daily distance of the mobility sub-model results (section 3) with the Swiss Mobility and Transport Microcensus 2010** (Bundesamt für Statistik (BFS) & Bundesamt für Raumentwicklung (ARE), 2012)**. [rel. Diff. = relative difference; abs. Diff. = absolute difference; PT = Public transport]**

	Mean daily distance (km/pers/day)	Microcensus	Model	rel. Diff.	abs. Diff.
Total	Total	36.7	37.1	0.011	0.4
Spatial Structures	Agglomeration (cores and isolated cities)	32.1	30.9	-0.037	-1.2
	Agglomeration (other municipalities)	37.0	37.2	0.005	0.2
	Rural municipalities	41.3	43.7	0.058	2.4
Household Types	1-Person HH	33.2	33.9	0.021	0.7
	2-Persons HH	37.2	36.3	-0.024	-0.9
	3-Persons HH	38.7	38.0	-0.018	-0.7
	4-Persons HH	36.6	38.5	0.052	1.9
	5+-Persons HH	37.0	38.8	0.049	1.8
Cities	Winterthur	37.8	39.4	0.042	1.6
	Zurich	35.6	35.2	-0.011	-0.4
	Bern	38.9	30.2	-0.224	-8.7
	Lucerne	37.2	33.2	-0.108	-4.0
	Lausanne	32.7	34.2	0.046	1.5
PT-classes	PT-class A	29.3	27.6	-0.058	-1.7
	PT-class B	31.3	32.2	0.029	0.9
	PT-class C	37.1	37.1	0.000	0.0
	PT-class D	39.1	41.3	0.056	2.2
	not classified	40.5	45.0	0.111	4.5

Table S1-6: **Comparison of the modal splits (based on kilometers driven) in the mobility sub-model (section 3) with the Swiss Mobility and Transport Microcensus 2010** (Bundesamt für Statistik (BFS) & Bundesamt für Raumentwicklung (ARE), 2012)**. [rel. Diff. = relative difference; abs. Diff. = absolute difference; PT = Public transport]**

	Modal splits (%)	Microcensus	Model	rel. Diff.	abs. Diff.
Total	Total: Car	73.9	72.4	-0.021	-1.6
	Total: PT	26.1	27.6	0.061	1.6
Household Types	1-Person HH: Car	67.0	64.0	-0.045	-3.0
	1-Person HH: PT	33.0	36.0	0.091	3.0
	2-Persons HH: Car	75.2	70.6	-0.062	-4.6
	2-Persons HH: PT	24.8	29.4	0.187	4.6
	3-Persons HH: Car	75.0	71.7	-0.045	-3.3
	3-Persons HH: PT	25.0	28.3	0.134	3.3
	4-Persons HH: Car	75.8	72.8	-0.039	-3.0
	4-Persons HH: PT	24.2	27.2	0.124	3.0
	5+-Persons HH: Car	69.8	72.5	0.038	2.7
	5+-Persons HH: PT	30.2	27.5	-0.088	-2.7
Cities	Winterthur: Car	56.0	67.0	0.198	11.1
	Winterthur: PT	44.0	33.0	-0.251	-11.1
	Zurich: Car	65.2	67.0	0.028	1.8
	Zurich: PT	34.8	33.0	-0.053	-1.8
	Bern: Car	64.9	69.9	0.077	5.0
	Bern: PT	35.1	30.1	-0.143	-5.0
	Lucerne: Car	67.7	78.3	0.157	10.6
	Lucerne: PT	32.3	21.7	-0.329	-10.6
	Lausanne: Car	79.1	74.9	-0.053	-4.2
	Lausanne: PT	20.9	25.1	0.201	4.2

6.2 Consumption Sub-Model in the Context of the Overall Model Compared to the Swiss Household Budget Survey

A selection of modeled revenues and expenditures are compared with the original data from the HBS, which can be regarded representative for Switzerland (Bundesamt für Statistik (BFS), 2013b), in Table S1-7 to Table S1-15. Deviations of more than 20% are indicated in red.

Table S1-7: Selection of modeled revenues and expenditures in the overall model compared with the original HBS-data (Bundesamt für Statistik (BFS), 2013b)**. Here, all households are included. [rel. Diff. = relative difference; abs. Diff. = absolute difference]**

(Swiss Francs per month)	All households			
	HBS	Model	rel. Diff.	abs. Diff.
Earned income	7227	7469	0.03	242
Primary income	7600	7880	0.04	280
Pensions and social benefits	1805	1757	-0.03	-48
Gross income	9530	9765	0.02	235
Compulsory transfer expenditure	2600	2704	0.04	104
Disposable income	6741	6876	0.02	135
Other insurances, fees and transfers	568	577	0.01	9
Total expenditures	5417	5550	0.02	133
Food and non-alcoholic beverages	654	662	0.01	7
Alcoholic beverages and tobacco	108	109	0.01	2
Restaurants and hotels	543	555	0.02	13
Clothing and footwear	234	239	0.02	5
Housing, water, electricity, gas and other fuels	1489	1540	0.03	50
Furnishings/household equip. and routine maintenance	277	285	0.03	7
Health	266	269	0.01	3
Transport	750	766	0.02	16
Communication	178	180	0.01	3
Recreation and culture	624	638	0.02	14
Miscellaneous goods and services	295	307	0.04	13

Table S1-8: **Selection of modeled revenues and expenditures in the overall model compared with the original HBS-data** (Bundesamt für Statistik (BFS), 2013b). **Here, the households are differentiated according to major regions (will be continued in Table S1-9). [rel. Diff. = relative difference; abs. Diff. = absolute difference]**

(Swiss Francs per month)	Major regions											
	Lake Geneva				Espace Mittelland				Northwestern Switzerland			
	HBS	Model	rel. Diff.	abs. Diff.	HBS	Model	rel. Diff.	abs. Diff.	HBS	Model	rel. Diff.	abs. Diff.
**Earned income**	7162	7268	0.01	107	6760	7326	0.08	566	7177	7608	0.06	431
**Primary income**	7557	7690	0.02	132	7045	7731	0.10	686	7511	8024	0.07	512
**Pensions and social benefits**	1975	1807	-0.08	-167	1832	1830	0.00	-2	1917	1742	-0.09	-176
**Gross income**	9632	9611	0.00	-21	8992	9693	0.08	701	9588	9897	0.03	309
**Compulsory transfer expenditure**	2844	2744	-0.04	-100	2548	2680	0.05	132	2610	2735	0.05	125
**Disposable income**	6629	6704	0.01	75	6208	6824	0.10	616	6793	6951	0.02	159
**Other insurances, fees and transfers**	594	577	-0.03	-17	571	579	0.01	8	536	578	0.08	42
**Total expenditures**	5451	5473	0.00	22	5132	5521	0.08	389	5348	5575	0.04	227
**Food and non-alcoholic beverages**	701	665	-0.05	-36	668	669	0.00	1	625	658	0.05	33
**Alcoholic beverages and tobacco**	117	110	-0.06	-7	102	109	0.07	7	106	107	0.01	1
**Restaurants and hotels**	501	516	0.03	15	508	550	0.08	41	533	563	0.06	30
**Clothing and footwear**	215	227	0.06	12	217	237	0.09	20	240	242	0.01	3
**Housing, water, electricity, gas and other fuels**	1538	1554	0.01	16	1361	1518	***0.11***	156	1471	1534	0.04	63
**Furnishings/household equip. and routine maintenance**	250	264	0.06	14	273	289	0.06	16	306	297	-0.03	-10
**Health**	291	279	-0.04	-12	261	272	0.04	11	248	263	0.06	16
**Transport**	735	754	0.03	19	736	754	0.02	18	730	780	0.07	50
**Communication**	199	189	-0.05	-10	171	175	0.02	4	166	176	0.06	10
**Recreation and culture**	626	615	-0.02	-11	583	643	***0.10***	60	626	644	0.03	18
**Miscellaneous goods and services**	278	298	0.07	20	250	306	***0.22***	56	296	310	0.05	14

Table S1-9: **Selection of modeled revenues and expenditures in the overall model compared with the original HBS-data** (Bundesamt für Statistik (BFS), 2013b). **Here, the households are differentiated according to major regions (continued from Table S1-8). [rel. Diff. = relative difference; abs. Diff. = absolute difference]**

(Swiss Francs per month)	Major regions															
	Zurich				Eastern Switzerland				Central Switzerland				Ticino			
	HBS	Model	rel. Diff.	abs. Diff.	HBS	Model	rel. Diff.	abs. Diff.	HBS	Model	rel. Diff.	abs. Diff.	HBS	Model	rel. Diff.	abs. Diff.
Earned income	7953	7851	-0.01	-103	7132	7533	0.06	401	7640	7713	0.01	73	6240	6335	0.02	95
Primary income	8407	8262	-0.02	-145	7471	7949	0.06	479	8095	8126	0.00	31	6638	6690	0.01	52
Pensions and social benefits	1860	1673	-0.10	-188	1485	1686	0.13	200	1571	1752	0.11	181	1813	1812	0.00	-1
Gross income	10380	10053	-0.03	-327	9086	9772	0.08	687	9814	10009	0.02	195	8594	8632	0.00	38
Compulsory transfer expenditure	2732	2752	0.01	20	2409	2693	0.12	284	2368	2748	0.16	380	2253	2314	0.03	62
Disposable income	7461	7111	-0.05	-349	6501	6889	0.06	388	7292	7095	-0.03	-197	6129	6166	0.01	36
Other insurances, fees and transfers	560	575	0.03	15	565	571	0.01	6	560	587	0.05	27	598	563	-0.06	-35
Total expenditures	5972	5704	-0.04	-268	5113	5532	0.08	419	5594	5701	0.02	108	5016	5039	0.00	23
Food and non-alcoholic beverages	621	642	0.03	21	644	666	0.03	22	659	691	0.05	32	636	625	-0.02	-11
Alcoholic beverages and tobacco	112	112	0.00	0	96	107	0.11	11	114	111	-0.02	-3	103	104	0.00	0
Restaurants and hotels	641	596	-0.07	-46	524	567	0.08	43	599	584	-0.02	-15	431	458	0.06	26
Clothing and footwear	262	250	-0.05	-12	234	242	0.03	8	248	250	0.01	1	229	219	-0.04	-10
Housing, water, electricity, gas and other fuels	1710	1594	-0.07	-116	1342	1519	0.13	177	1533	1559	0.02	26	1358	1415	0.04	58
Furnishings/household equip. and routine maintenance	296	296	0.00	0	266	283	0.06	17	294	293	0.00	-1	248	251	0.01	2
Health	287	263	-0.08	-24	240	264	0.10	24	250	275	0.10	26	251	250	-0.01	-2
Transport	782	790	0.01	7	724	754	0.04	30	810	784	-0.03	-26	752	726	-0.03	-25
Communication	179	182	0.02	3	165	177	0.07	12	169	178	0.06	10	201	188	-0.06	-12
Recreation and culture	693	657	-0.05	-35	609	645	0.06	37	625	662	0.06	36	555	539	-0.03	-16
Miscellaneous goods and services	389	323	-0.17	-66	269	308	0.14	39	293	314	0.07	21	251	265	0.05	13

Table S1-10: **Selection of modeled revenues and expenditures in the overall model compared with the original HBS-data** (Bundesamt für Statistik (BFS), 2013b). **Here, the households are differentiated according to largest cantons (will be continued in Table S1-11). [rel. Diff. = relative difference; abs. Diff. = absolute difference]**

(Swiss Francs per month)	Largest cantons															
	Zurich				Bern				Lucerne				St. Gallen			
	HBS	Model	rel. Diff.	abs. Diff.	HBS	Model	rel. Diff.	abs. Diff.	HBS	Model	rel. Diff.	abs. Diff.	HBS	Model	rel. Diff.	abs. Diff.
Earned income	7953	7851	-0.01	-103	6503	7228	0.11	725	6975	7673	0.10	698	7196	7542	0.05	345
Primary income	8407	8262	-0.02	-145	6837	7635	0.12	798	7333	8082	0.10	750	7575	7954	0.05	379
Pensions and social benefits	1860	1673	-0.10	-188	1849	1859	0.01	10	1516	1753	0.16	237	1467	1671	0.14	204
Gross income	10380	10053	-0.03	-327	8797	9622	0.09	825	9045	9966	0.10	921	9183	9764	0.06	581
Compulsory transfer expenditure	2732	2752	0.01	20	2518	2662	0.06	144	2219	2731	***0.23***	512	2449	2689	0.10	239
Disposable income	7461	7111	-0.05	-349	6013	6771	0.13	758	6672	7071	0.06	399	6562	6886	0.05	324
Other insurances, fees and transfers	560	575	0.03	15	569	577	0.01	8	543	585	0.08	42	567	570	0.00	3
Total expenditures	5972	5704	-0.04	-268	5004	5485	0.10	481	5354	5681	0.06	327	5167	5528	0.07	361
Food and non-alcoholic beverages	621	642	0.03	21	643	664	0.03	21	645	689	0.07	44	623	665	0.07	42
Alcoholic beverages and tobacco	112	112	0.00	0	98	109	0.11	11	100	110	0.10	10	92	107	0.16	15
Restaurants and hotels	641	596	-0.07	-46	507	546	0.08	39	594	582	-0.02	-11	528	567	0.07	39
Clothing and footwear	262	250	-0.05	-12	212	234	0.11	23	251	249	-0.01	-2	236	242	0.02	6
Housing, water, electricity, gas and other fuels	1710	1594	-0.07	-116	1349	1511	0.12	162	1483	1556	0.05	73	1379	1519	0.10	140
Furnishings/household equip. and routine maintenance	296	296	0.00	0	260	287	0.10	27	259	292	0.13	33	258	282	0.09	24
Health	287	263	-0.08	-24	266	272	0.02	5	243	274	0.13	31	213	263	***0.24***	50
Transport	782	790	0.01	7	661	747	0.13	86	722	780	0.08	58	767	755	-0.02	-13
Communication	179	182	0.02	3	167	173	0.04	6	157	178	0.13	20	168	178	0.06	10
Recreation and culture	693	657	-0.05	-35	584	639	0.09	55	616	659	0.07	43	621	644	0.04	23
Miscellaneous goods and services	389	323	-0.17	-66	258	304	0.18	46	284	312	0.10	28	283	307	0.09	24

Table S1-11: **Selection of modeled revenues and expenditures in the overall model compared with the original HBS-data** (Bundesamt für Statistik (BFS), 2013b). **Here, the households are differentiated according to largest cantons (continued from Table S1-10). [rel. Diff. = relative difference; abs. Diff. = absolute difference]**

(Swiss Francs per month)	Largest cantons															
	Aargau				Ticino				Vaud				Geneva			
	HBS	Model	rel. Diff.	abs. Diff.	HBS	Model	rel. Diff.	abs. Diff.	HBS	Model	rel. Diff.	abs. Diff.	HBS	Model	rel. Diff.	abs. Diff.
**Earned income**	7789	7836	0.01	48	6240	6335	0.02	95	7338	7299	-0.01	-39	7487	7166	-0.04	-321
**Primary income**	8117	8252	0.02	136	6638	6690	0.01	52	7763	7718	-0.01	-46	7913	7579	-0.04	-334
**Pensions and social benefits**	1614	1706	0.06	92	1813	1812	0.00	-1	1980	1807	-0.09	-173	2037	1748	-0.14	-288
**Gross income**	9862	10090	0.02	227	8594	8632	0.00	38	9867	9639	-0.02	-228	10020	9442	-0.06	-578
**Compulsory transfer expenditure**	2643	2791	0.06	148	2253	2314	0.03	62	2838	2754	-0.03	-84	3157	2696	-0.15	-461
**Disposable income**	7024	7090	0.01	66	6129	6166	0.01	36	6893	6721	-0.02	-172	6708	6592	-0.02	-116
**Other insurances, fees and transfers**	543	586	0.08	43	598	563	-0.06	-35	595	579	-0.03	-15	592	560	-0.05	-32
**Total expenditures**	5567	5675	0.02	108	5016	5039	0.00	23	5465	5487	0.00	23	5836	5372	-0.08	-465
**Food and non-alcoholic beverages**	646	672	0.04	26	636	625	-0.02	-11	691	668	-0.03	-22	704	644	-0.08	-60
**Alcoholic beverages and tobacco**	108	109	0.00	0	103	104	0.00	0	125	111	-0.12	-15	111	107	-0.04	-5
**Restaurants and hotels**	547	575	0.05	28	431	458	0.06	26	487	518	0.06	31	566	505	-0.11	-61
**Clothing and footwear**	254	248	-0.02	-6	229	219	-0.04	-10	210	228	0.08	18	230	221	-0.04	-9
**Housing, water, electricity, gas and other fuels**	1463	1548	0.06	85	1358	1415	0.04	58	1589	1556	-0.02	-34	1724	1548	-0.10	-175
**Furnishings/household equip. and routine maintenance**	346	304	-0.12	-41	248	251	0.01	2	232	265	0.14	33	238	255	0.07	17
**Health**	237	267	0.12	30	251	250	-0.01	-2	266	279	0.05	13	378	274	***-0.28***	-104
**Transport**	860	798	-0.07	-62	752	726	-0.03	-25	793	759	-0.04	-35	639	736	0.15	97
**Communication**	170	179	0.05	9	201	188	-0.06	-12	193	190	-0.02	-4	224	190	-0.15	-34
**Recreation and culture**	653	659	0.01	6	555	539	-0.03	-16	626	617	-0.02	-10	674	600	-0.11	-74
**Miscellaneous goods and services**	282	316	0.12	34	251	265	0.05	13	251	298	0.19	47	349	292	-0.16	-56

Table S1-12: **Selection of modeled revenues and expenditures in the overall model compared with the original HBS-data** (Bundesamt für Statistik (BFS), 2013b). **Here, the households are differentiated according to income classes (will be continued in Table S1-13). [rel. Diff. = relative difference; abs. Diff. = absolute difference]**

(Swiss Francs per month)	Income classes (Swiss Francs per month)											
	< 4880				4880 - 7173				7174 - 9702			
	HBS	Model	rel. Diff.	abs. Diff.	HBS	Model	rel. Diff.	abs. Diff.	HBS	Model	rel. Diff.	abs. Diff.
**Earned income**	904	212	***-0.77***	-692	3540	3845	0.09	304	6147	6417	0.04	270
**Primary income**	1072	685	***-0.36***	-386	3767	4098	0.09	331	6367	6681	0.05	314
**Pensions and social benefits**	2270	3736	***0.65***	1466	2153	2331	0.08	178	1941	1736	-0.11	-206
**Gross income**	3475	4527	***0.30***	1052	6053	6523	0.08	471	8414	8630	0.03	216
**Compulsory transfer expenditure**	865	1024	0.18	159	1542	1669	0.08	127	2188	2271	0.04	83
**Disposable income**	2538	3212	***0.27***	674	4299	4752	0.11	453	6074	6246	0.03	171
**Other insurances, fees and transfers**	315	423	***0.34***	107	446	436	-0.02	-11	552	527	-0.05	-25
**Total expenditures**	3038	3067	0.01	28	4089	3993	-0.02	-95	5105	5154	0.01	49
**Food and non-alcoholic beverages**	444	410	-0.08	-34	530	470	-0.11	-60	638	638	0.00	1
**Alcoholic beverages and tobacco**	68	52	***-0.24***	-16	81	91	0.12	9	100	101	0.01	1
**Restaurants and hotels**	226	191	-0.16	-35	358	393	0.10	35	495	474	-0.04	-21
**Clothing and footwear**	96	106	0.10	9	150	145	-0.03	-5	197	213	0.08	16
**Housing, water, electricity, gas and other fuels**	1061	1156	0.09	95	1266	1281	0.01	15	1448	1521	0.05	73
**Furnishings/household equip. and routine maintenance**	120	150	***0.25***	30	185	159	-0.14	-26	253	263	0.04	10
**Health**	194	213	0.10	19	234	245	0.05	11	266	245	-0.08	-21
**Transport**	298	232	***-0.22***	-66	528	469	-0.11	-59	701	728	0.04	28
**Communication**	109	78	***-0.29***	-31	152	142	-0.07	-10	181	186	0.03	5
**Recreation and culture**	294	318	0.08	24	418	426	0.02	8	574	558	-0.03	-15
**Miscellaneous goods and services**	127	161	***0.26***	33	186	172	-0.08	-14	253	225	-0.11	-27

Table S1-13: **Selection of modeled revenues and expenditures in the overall model compared with the original HBS-data** (Bundesamt für Statistik (BFS), 2013b). **Here, the households are differentiated according to income classes (continued from Table S1-12). [rel. Diff. = relative difference; abs. Diff. = absolute difference]**

(Swiss Francs per month)	Income classes (Swiss Francs per month)							
	9703 - 13170				> 13171			
	HBS	Model	rel. Diff.	abs. Diff.	HBS	Model	rel. Diff.	abs. Diff.
Earned income	9443	10626	0.13	1183	16099	14633	-0.09	-1466
Primary income	9718	10850	0.12	1131	17074	15713	-0.08	-1361
Pensions and social benefits	1441	933	*-0.35*	-509	1221	923	*-0.24*	-297
Gross income	11255	11862	0.05	606	18448	16750	-0.09	-1698
Compulsory transfer expenditure	2957	3114	0.05	158	5447	5335	-0.02	-112
Disposable income	8108	8377	0.03	269	12682	11241	-0.11	-1442
Other insurances, fees and transfers	630	645	0.02	15	897	876	-0.02	-20
Total expenditures	6246	6531	0.05	284	8604	8697	0.01	93
Food and non-alcoholic beverages	768	777	0.01	9	891	994	0.12	103
Alcoholic beverages and tobacco	129	128	-0.01	-1	159	155	-0.02	-4
Restaurants and hotels	659	706	0.07	48	975	932	-0.04	-43
Clothing and footwear	290	296	0.02	6	436	429	-0.01	-7
Housing, water, electricity, gas and other fuels	1611	1730	0.07	119	2061	1950	-0.05	-110
Furnishings/household equip. and routine maintenance	307	323	0.05	15	522	542	0.04	20
Health	290	257	-0.11	-33	344	380	0.10	36
Transport	942	1006	0.07	65	1279	1273	-0.01	-7
Communication	210	217	0.03	7	237	239	0.01	2
Recreation and culture	722	765	0.06	43	1113	1107	-0.01	-7
Miscellaneous goods and services	319	326	0.02	7	588	697	0.18	109

Table S1-14: **Selection of modeled revenues and expenditures in the overall model compared with the original HBS-data** (Bundesamt für Statistik (BFS), 2013b). **Here, the households are differentiated according to household types (will be continued in Table S1-15). [rel. Diff. = relative difference; abs. Diff. = absolute difference]**

(Swiss Francs per month)	All 1-person households				All couples with children			
	HBS	Model	rel. Diff.	abs. Diff.	HBS	Model	rel. Diff.	abs. Diff.
Earned income	3899	5715	*0.47*	1816	10770	9633	-0.11	-1136
Primary income	4254	6071	*0.43*	1817	11004	9967	-0.09	-1036
Pensions and social benefits	1815	1658	-0.09	-157	780	1109	*0.42*	329
Gross income	6156	7843	*0.27*	1687	11890	11243	-0.05	-647
Compulsory transfer expenditure	1620	2134	*0.32*	514	3291	3115	-0.05	-176
Disposable income	4273	5490	*0.28*	1217	8510	7999	-0.06	-511
Other insurances, fees and transfers	419	470	0.12	51	622	616	-0.01	-6
Total expenditures	3696	4510	*0.22*	815	6868	6372	-0.07	-496
Food and non-alcoholic beverages	378	488	*0.29*	110	909	816	-0.10	-93
Alcoholic beverages and tobacco	75	88	0.18	13	101	110	0.08	8
Restaurants and hotels	365	445	*0.22*	80	654	630	-0.04	-24
Clothing and footwear	136	181	*0.33*	45	338	300	-0.11	-38
Housing, water, electricity, gas and other fuels	1249	1411	0.13	163	1772	1679	-0.05	-93
Furnishings/household equip. and routine maintenance	155	207	*0.33*	51	362	343	-0.05	-19
Health	181	209	0.15	28	279	271	-0.03	-8
Transport	458	593	*0.29*	135	968	892	-0.08	-76
Communication	124	156	*0.26*	32	226	210	-0.07	-16
Recreation and culture	400	499	*0.25*	99	815	742	-0.09	-73
Miscellaneous goods and services	174	233	*0.34*	59	444	379	-0.15	-65

Table S1-15: **Selection of modeled revenues and expenditures in the overall model compared with the original HBS-data** (Bundesamt für Statistik (BFS), 2013b). **Here, the households are differentiated according to household types (continued from Table S1-14). [rel. Diff. = relative difference; abs. Diff. = absolute difference]**

(Swiss Francs per month)	Household types											
	Couples with 1 child				Couples with 2 children				Couples with 3+ children			
	HBS	Model	rel. Diff.	abs. Diff.	HBS	Model	rel. Diff.	abs. Diff.	HBS	Model	rel. Diff.	abs. Diff.
**Earned income**	10250	10253	0.00	3	11124	10253	-0.08	-872	11019	10519	-0.05	-501
**Primary income**	10448	10598	0.01	150	11359	10598	-0.07	-761	11337	10882	-0.04	-455
**Pensions and social benefits**	693	1108	***0.60***	415	752	1108	***0.47***	356	1057	1107	0.05	50
**Gross income**	11215	11886	0.06	672	12238	11886	-0.03	-352	12522	12175	-0.03	-347
**Compulsory transfer expenditure**	3213	3277	0.02	64	3323	3277	-0.01	-46	3383	3343	-0.01	-40
**Disposable income**	7893	8482	0.07	589	8823	8482	-0.04	-341	9099	8707	-0.04	-393
**Other insurances, fees and transfers**	577	652	0.13	74	660	652	-0.01	-9	622	667	0.07	44
**Total expenditures**	6440	6735	0.05	295	7131	6735	-0.06	-396	7153	6905	-0.03	-249
**Food and non-alcoholic beverages**	779	891	0.14	112	953	891	-0.07	-63	1089	925	-0.15	-164
**Alcoholic beverages and tobacco**	109	116	0.07	7	105	116	0.11	12	74	118	***0.60***	44
**Restaurants and hotels**	618	677	0.09	58	706	677	-0.04	-29	599	701	0.17	102
**Clothing and footwear**	297	325	0.10	29	365	325	-0.11	-40	359	337	-0.06	-22
**Housing, water, electricity, gas and other fuels**	1738	1686	-0.03	-52	1810	1686	-0.07	-124	1748	1684	-0.04	-63
**Furnishings/household equip. and routine maintenance**	336	362	0.08	26	361	362	0.00	1	426	374	-0.12	-52
**Health**	249	297	0.19	48	298	297	0.00	-1	296	309	0.04	13
**Transport**	1011	955	-0.06	-56	950	955	0.00	4	917	983	0.07	67
**Communication**	228	221	-0.03	-7	223	221	-0.01	-2	227	223	-0.02	-4
**Recreation and culture**	619	811	***0.31***	192	934	811	-0.13	-123	951	849	-0.11	-101
**Miscellaneous goods and services**	456	394	-0.14	-62	426	394	-0.07	-32	468	401	-0.14	-67

Table S1-7 to Table S1-15 show that the model is able to satisfactorily reproduce the statistics according to the HBS in different aggregated forms, be it grouped by geographical major regions, individual cantons, income classes or household types. Among all these comparisons, only two tables exhibit larger deviations: the income class "< 4880 Swiss Francs per month" in Table S1-12 and "1-person households" in Table S1-14. Although these deviations are still acceptable, they are also explainable. In fact, only one archetype (cluster H, the "old, widowed females") shows an income lower than 4880 Swiss Francs per month (see (Froemelt et al., 2018)). This indicates that only one specific archetype was considered in this income class and deviations were clearly to be expected. In other words: the official income class subdivision of the HBS is not sensible for comparing with our model (at least not for comparing low-income classes). However, the case is different for single-person households. Here, the reasons for the deviations lie in the averaging procedure that was applied to derive the archetypes and in the classification approach to assign the archetypes to households. In fact, none of the archetypes is a "pure" single-person household (average number of persons equals exactly 1). Therefore, larger households in the same cluster might tend to increase the average values of "almost" single-person household archetypes. Indeed, Table S1-14 shows overestimations by trend.

6.3 Comparison with Further National Statistics

Table S1-16 compares the overall model's results to further national statistics of Switzerland: Final energy demand for fuel oil, electricity as well as total energy demand for heating correspond well with national statistics (Bundesamt für Energie (BFE), 2016). The modeled water consumption is higher than the current direct water consumption according to (Schweizerischer Verein des Gas- und Wasserfaches (SVGW), 2017). However, the statistics here are not measured values but an estimation itself and it does not refer to a specific year. Therefore, the model results can still be regarded as a reasonable estimate. The computed average waste production of 193 kg/pers/yr is clearly below the waste statistics of 344 kg/pers/yr, but still in the same order of magnitude. The underestimation of the model can partly be explained by the fact that the waste statistics (Bundesamt für Umwelt (BAFU), 2014) takes not only household waste but also commercial waste into account. Nevertheless, a rough sensitivity analysis revealed that the final life cycle assessment results are only negligibly affected even in the case of doubling the waste production.

Table S1-16: **Comparison of model results with national statistics. Final energy demand statistics originate from** (Bundesamt für Energie (BFE), 2016) **and refer to the year 2013, while water consumption was retrieved from** (Schweizerischer Verein des Gas- und Wasserfaches (SVGW), 2017) **and waste statistics from** (Bundesamt für Umwelt (BAFU), 2014)**.**

	Statistics	Model
**Total final energy for heating[a] (GJ/pers/yr)**	24.1	27.2
**Final energy for heating (only fuel oil) (GJ/pers/yr)**	12.5	14.7
**Final energy for electricity (kWh/pers/yr)**	2365	2528
**Water consumption (m^3/pers/yr)**	52	69
**Waste production (kg/pers/yr)**	344	193

[a]Electric heating systems excluded

6.4 Comparison with Further Income Statistics

As mentioned in the main body of the article, we used the modeled incomes of the households as additional variables in the status quo analysis (see section 7) because official external municipal income statistics do not exist. For this reason, we laid a special focus on the evaluation of the modeled incomes, particularly with regard to its spatial distribution. First, we would like to point out the comparisons performed in Table S1-8 to Table S1-11. There, it shows that the model is able to satisfactorily reproduce the average earned, primary and gross income for the seven major regions in Switzerland as well as for the eight largest cantons. In addition to these comparisons, we also exposed our model results to a completely independent income-dataset in Table S1-17. In this table, we compare the median of the major regions' so-called equalized disposable income with the Swiss SILC-survey (Statistics on Income and Living Conditions, (Bundesamt für Statistik (BFS), 2017)). The equalized disposable income is computed by division of the disposable income by the equalized household size that is given by the "OECD-modified scale" and that "*assigns a value of 1 to the household head, of 0.5 to each additional adult member and of 0.3 to each child*" (OECD, 2019). As can be seen in Table S1-17, we compare our model results to a minimum and a maximum value of SILC. The reason for this lies in the different reference years: While our overall model refers to 2013, the underlying archetypes source HBS-data from 2009 to 2011. Therefore, we extracted the minimum and maximum values from the SILC-datasets for these four years. However, the range is rather narrow and the model results lie either within the minimum and maximum values or deviate by a maximum of 0.7%. The Pearson correlation coefficient for the model results with SILC-Max amounts to 0.95 and to 0.82 with SILC-Min, respectively.

Table S1-17: Comparison of the median equalized disposable income for the seven major regions of Switzerland and the SILC-datasets of 2009-2011 and 2013 (Bundesamt für Statistik (BFS), 2017)**. Overshoots of Max (SILC) are indicated in red, while undershoots of Min (SILC) are shown in blue.**

	Median equalized disposable income (CHF/yr)			
**Major Regions**	**Max (SILC)**	**Model**	**Min (SILC)**	**rel. Diff.**
**Lake Geneva**	49720	49918	47152	0.004
**Espace Mittelland**	47786	46009	43419	
**Northwestern Switzerland**	54772	52874	49622	
**Zurich**	56549	54541	53064	
**Eastern Switzerland**	50166	50133	44060	
**Central Switzerland**	52907	53279	46640	0.007
**Ticino**	45806	42721	42988	-0.067

7 Status Quo Analysis

As mentioned in the main body of the article, we grouped all 2352 Swiss municipalities into four clusters according to their average municipal carbon footprint for the status quo analysis: Cluster LO comprises the 10% of the municipalities with the lowest emissions (6.0 – 8.7 t CO_2-eq per person per year), low-to-medium-emission municipalities belong to cluster LM (10% – 50%-percentiles, 8.7 – 9.8 t CO_2-eq per person per year), cluster MH stands for medium-to-high communities (50% – 90%-percentiles, 9.8 – 11.3 t CO_2-eq per person per year) and the 10% highest emitting municipalities are member of cluster HI (11.3 – 16.6 t CO_2-eq per person per year). The subdivision of municipalities is also depicted in Figure S1-5.

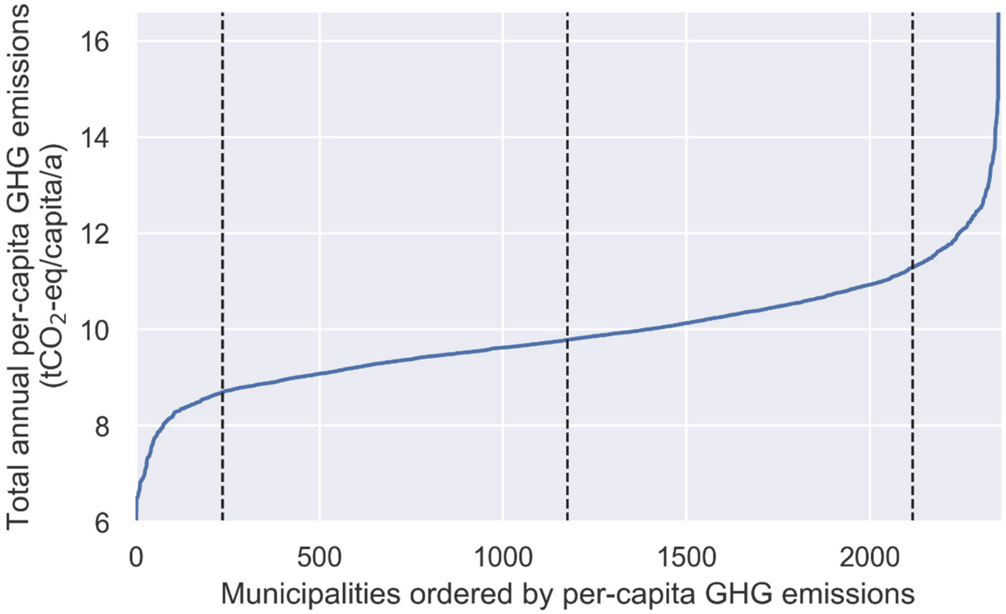

Figure S1-5: Subdivision of municipalities for the status quo analysis according to their per-capita greenhouse gas (GHG) emissions. The borders correspond to the 10%-, 50%- and 90%-percentiles.

In a next step, continuous variables and categorical variables representing municipal characteristics were investigated. Apart from a visual judgement, we also applied a one-way ANOVA-test to the continuous variables (Fisher, 1973). The resulting test statistics (F-value) for a certain variable can be regarded as a quantitative measure of how much the four municipal clusters are distinct in the variable under consideration. In other words: the larger the F-value, the more the clusters differ from each other with regard to this variable. Continuous variables are presented in Figure S1-6 to Figure S1-9 and comprise the following:

- No. of persons / households per municipality
- Model results for main consumption areas (in kgCO_2-eq per person per year)
- Statistics on household size structure (as a share of a certain household size type in all households of the municipality). The subdivision of household size types correspond to the official statistics in (Bundesamt für Statistik (BFS), 2014c).
- Statistics on household age structure (as a share of a certain household age type in all households of the municipality). The subdivision of household age types correspond to the official statistics in (Bundesamt für Statistik (BFS), 2014c).

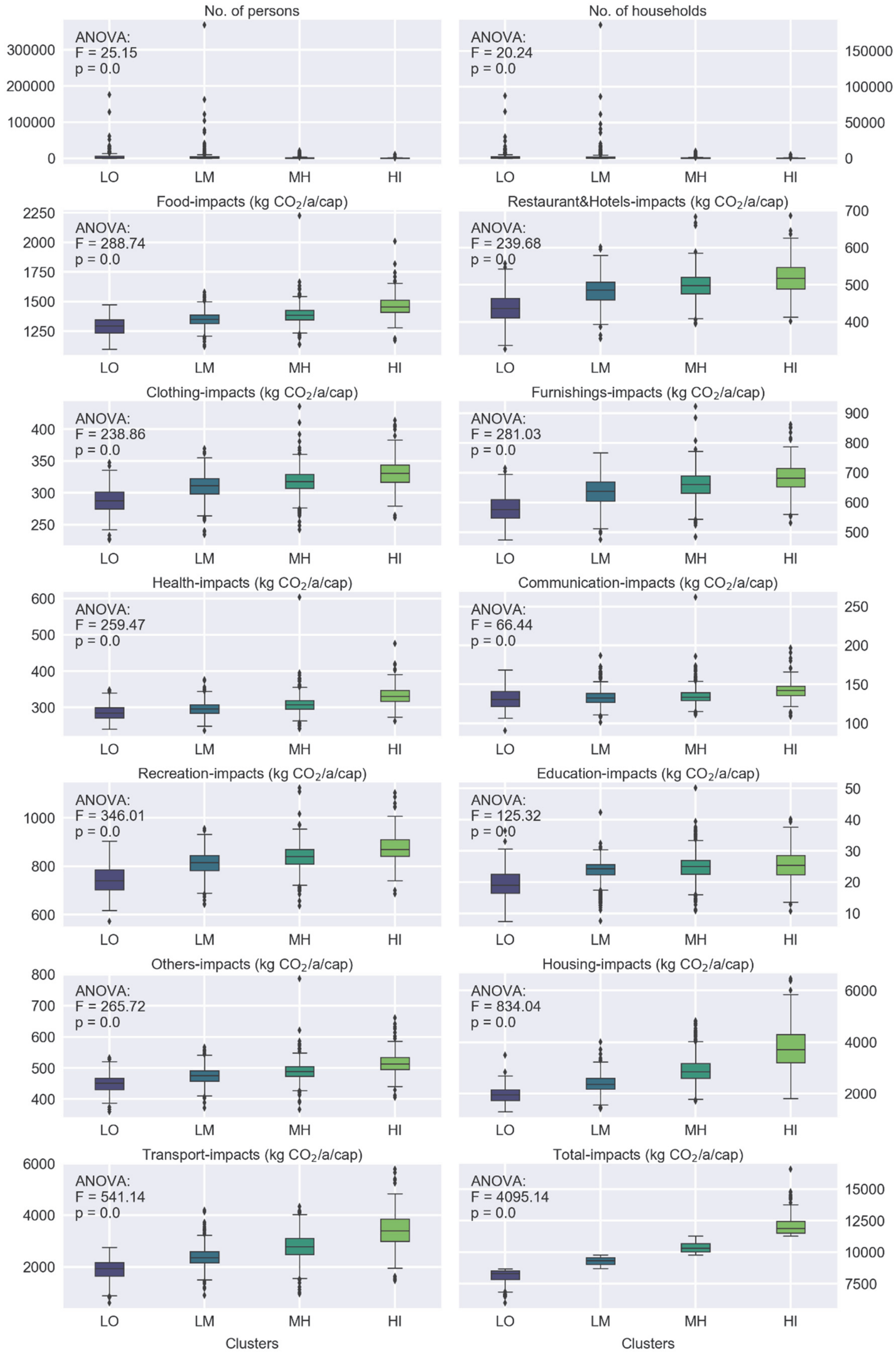

Figure S1-6: **Distribution of municipal statistics (continuous variables) for the four different municipal clusters. See text for more information. Note that some of the y-axes do not start at zero.**

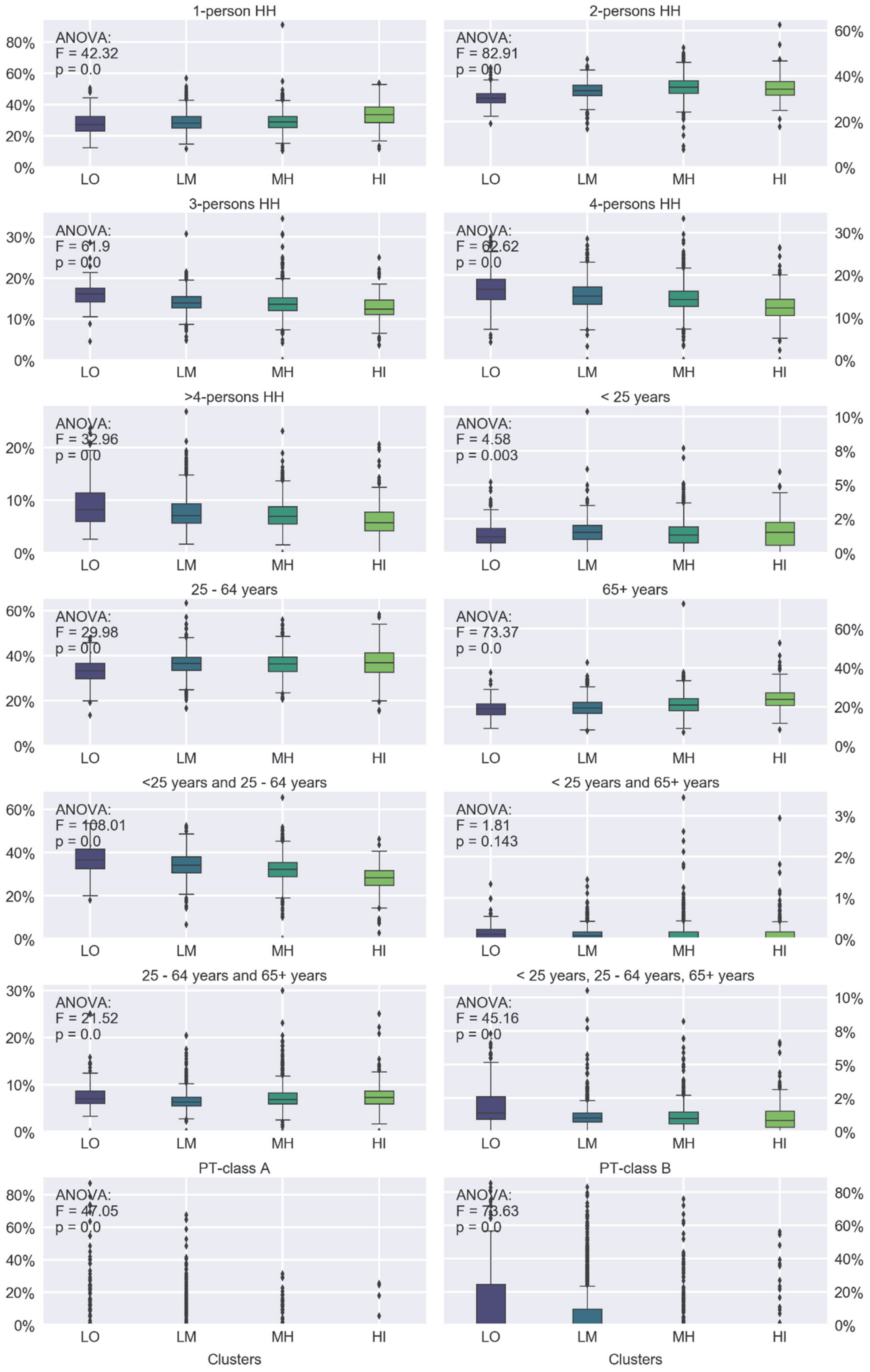

Figure S1-7: **Distribution of municipal statistics (continuous variables) for the four different municipal clusters. See text for more information.**

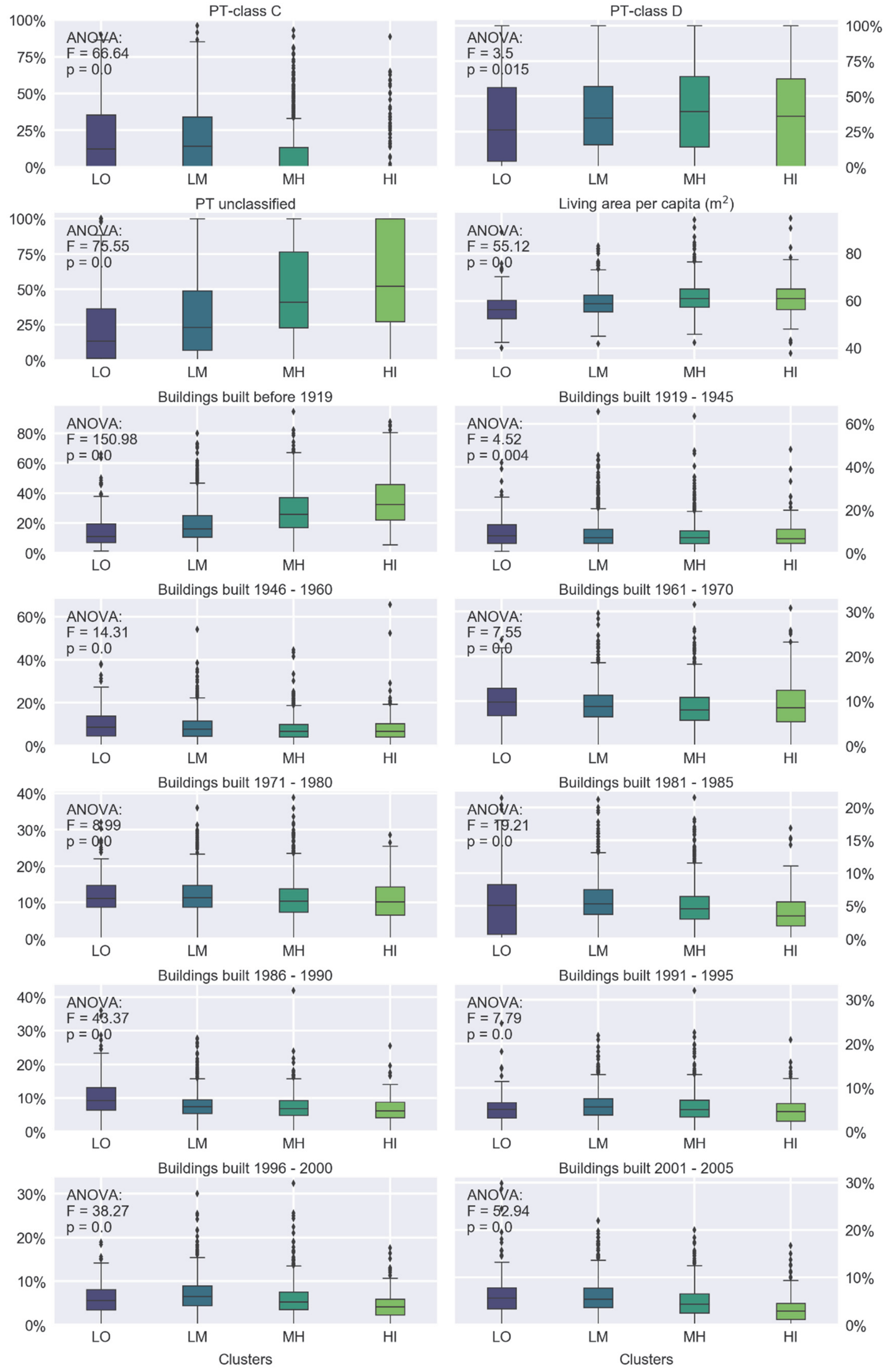

Figure S1-8: **Distribution of municipal statistics (continuous variables) for the four different municipal clusters. See text for more information.**

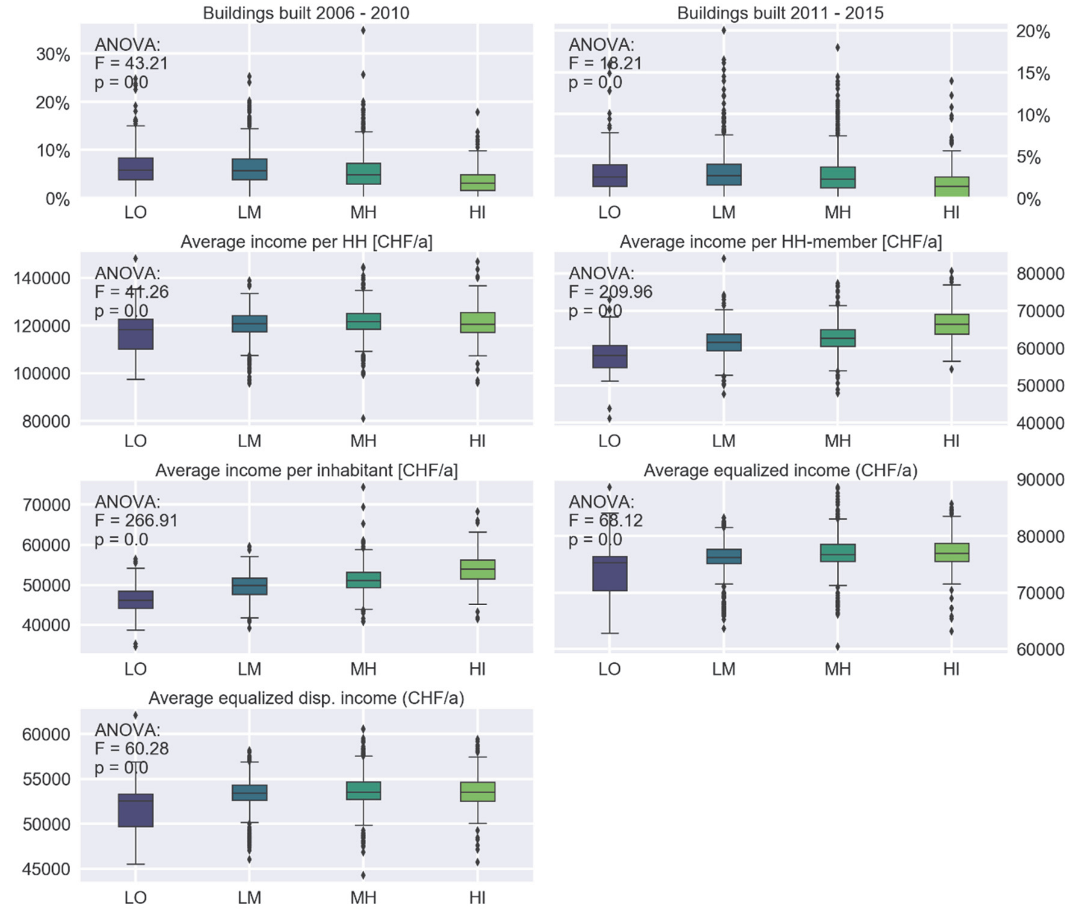

Figure S1-9: **Distribution of municipal statistics (continuous variables) for the four different municipal clusters. See text for more information.**

- Public transport services as share of people living in a certain PT-class (ARE (Bundesamt für Raumentwicklung), 2017) (see also section 6.1 for more explanations)
- Living area per capita (in m^2 per person)
- Building stock statistics as shares of buildings of a certain construction period in all buildings of the municipality. The construction periods correspond to the official subdivision in (Bundesamt für Statistik (BFS), 2014b).
- Modeled incomes as average income per household, per household member as well as per inhabitant. Additionally, the equalized average income per household and the equalized disposable income per household are displayed (see section 6.4 for a description to compute the equalized incomes).

Figure S-10 to Figure S1-11 look at categorical classifications of municipalities (i.e. at attributes that are valid for the whole municipality). These encompass the DEGURBA-classification ("Degree of Urbanisation"-definition of the European Union which classifies municipalities into "thinly populated area", "intermediate density area", and "densely populated area", (Eurostat, 2019)), the classification into rural, urban cores or agglomeration communities by the Federal Statistical Office (Bundesamt für Statistik (BFS), 2014a) as well as Swiss major regions. In Figure S1-10-Figure S1-11, different perspectives on these statistics are provided. Figure S1-10 weighs the statistics by the number of persons living in the municipalities, while Figure S1-11 considers the count of municipalities in a certain classification. In both figures, the left-hand side looks at how the categorical variables are distributed among the clusters, whereas the

right-hand side presents the composition of the clusters. The following reading examples apply:

- Figure S1-10, left (distribution among clusters): "Of all people living in densely populated areas, how many live in cluster LO, LM, MH, HI."
- Figure S1-10, right (composition of clusters): "Of all people living in municipalities classified as LO, how many are living in densely populated, intermediate populated or thinly populated areas."
- Figure S1-11, left (distribution among clusters): "Of all municipalities classified as densely populated areas, how many were allocated to the clusters LO, LM, MH, HI."
- Figure S1-11, right (composition of clusters): "Of all municipalities classified as LO, how many are attributed as densely populated, intermediate populated or thinly populated areas."

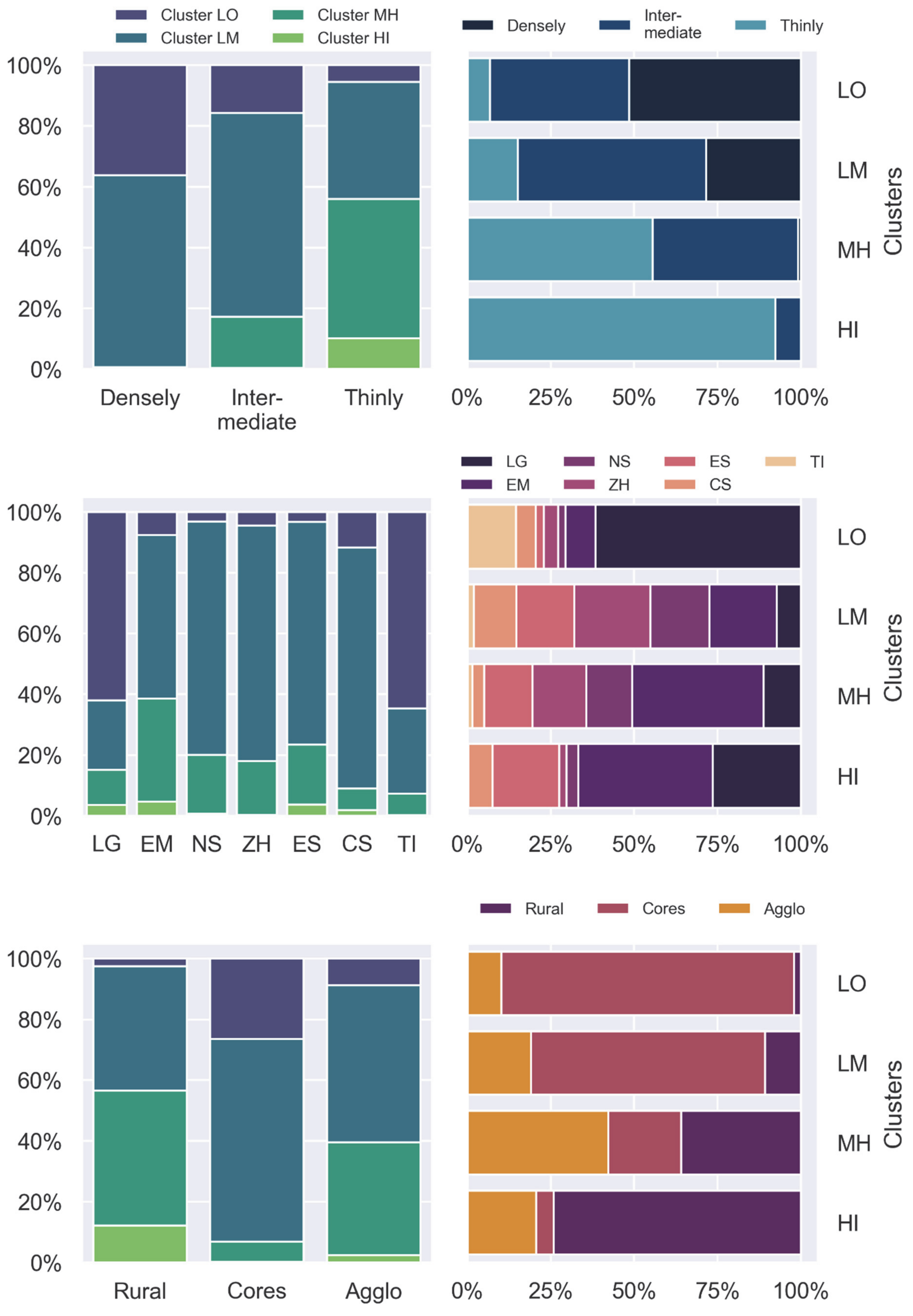

Figure S1-10: **Left: Distribution of persons living in a certain categorized area among the municipal clusters; Right: Composition of the municipal clusters according to people living in differently classified areas. See text for more information and reading examples. [LG = Lake Geneva; EM = Espace Mittelland; NS = Northwestern Switzerland; ZH = Zurich; ES = Eastern Switzerland; CS = Central Switzerland; TI = Ticino]**

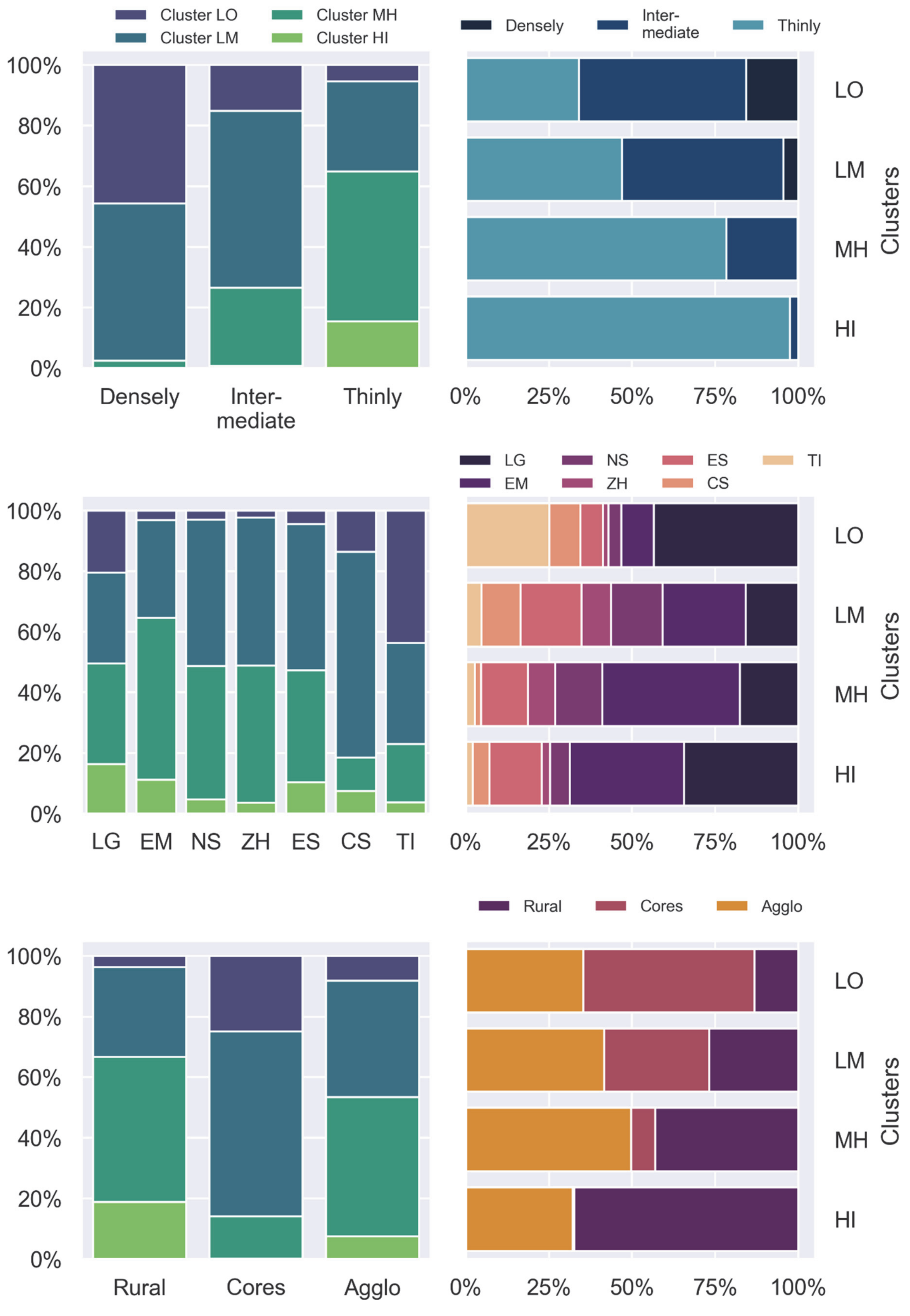

Figure S1-11: **Left: Distribution of municipalities of a certain category among the municipal clusters; Right: Composition of the municipal clusters according to number of municipalities of different categories. See text for more information and reading examples. [LG = Lake Geneva; EM = Espace Mittelland; NS = Northwestern Switzerland; ZH = Zurich; ES = Eastern Switzerland; CS = Central Switzerland; TI = Ticino]**

References

ARE (Bundesamt für Raumentwicklung). (2017). *ÖV-Güteklassen. Berechnungsmethodik ARE. Grundlagenbericht für die Beurteilung der Agglomerationsprogramme Verkehr und Siedlung*.

Breiman, L. (2001). Random Forests. *Machine Learning*, *45*(1), 5–32. https://doi.org/10.1023/A:1010933404324

Breiman, L., & Cutler, A. (2003). *Manual on Setting Up, Using, and Understanding Random Forests V4.0*. https://doi.org/10.1017/CBO9781107415324.004

Bundesamt für Energie (BFE). (2015). *Schweizerische Gesamtenergiestatistik 2014*. Bern-Ittigen.

Bundesamt für Energie (BFE). (2016). *Schweizerische Gesamtenergiestatistik 2015*. Bundesamt für Energie (BFE): Bern-Ittigen, Switzerland.

Bundesamt für Statistik (BFS). *Eidgenössisches Gebäude- und Wohnungsregister*. , (2013).

Bundesamt für Statistik (BFS). *Haushaltsbudgeterhebung (HABE) 2009 bis 2011*. , (2013).

Bundesamt für Statistik (BFS). (2014a). *Die Raumgliederungen der Schweiz 2014*. Neuchâtel, Switzerland, Fed Regist.

Bundesamt für Statistik (BFS). (2014b). *GWS 2013 - Gebäude- und Wohnungsstatistik*. Neuchâtel, Switzerland, Fed Regist.

Bundesamt für Statistik (BFS). (2014c). *STATPOP 2013 - Statistik der Bevölkerung und Haushalte*. Neuchâtel, Switzerland, Fed Regist.

Bundesamt für Statistik (BFS). (2014d). *Strassenfahrzeugbestand 2013*. Neuchâtel, Switzerland.

Bundesamt für Statistik (BFS). *Erhebung über die Einkommen und Lebensbedingungen (SILC)*. , (2017).

Bundesamt für Statistik (BFS), & Bundesamt für Raumentwicklung (ARE). (2012). *Mobilität in der Schweiz. Ergebnisse des Mikrozensus Mobilität und Verkehr 2010*. Neuchâtel, Switzerland: Bundesamt für Statistik (BFS).

Bundesamt für Umwelt (BAFU). (2012). Kommunale Abwasserreinigung. Retrieved January 20, 2017, from https://www.bafu.admin.ch/bafu/de/home/themen/wasser/fachinformationen/massnahmen-zum-schutz-der-gewaesser/abwasserreinigung/kommunale-abwasserreinigung.html

Bundesamt für Umwelt (BAFU). (2014). *Abfallmengen und Recycling 2013 im Überblick*. Bundesamt für Umwelt (BAFU): Bern, Switzerland.

Ecoinvent Centre. (2013). *Ecoinvent data, version 2.2*. 1998-2013: http://www.ecoinvent.org/database/.

Ecoinvent Centre. (2016). Ecoinvent database (Version 3.3). [Cut-off system model]. Retrieved from www.ecoinvent.org. Retrieved July 29, 2016, from www.ecoinvent.org

Eurostat. (2019). Degree of Urbanisation (DEGURBA). Retrieved February 14, 2019, from https://ec.europa.eu/eurostat/web/gisco/geodata/reference-data/population-distribution-demography/degurba

EXIOBASE Consortium. (2014). EXIOBASE (Version 2.2.2). Retrieved from EXIOBASE. Retrieved January 12, 2018, from https://www.exiobase.eu/index.php/data-download/exiobase2-year-2007-full-data-set/79-mriot-pxp-ita-coefficient-version2-2-2.

Fernández-Delgado, M., Cernadas, E., Barro, S., & Amorim, D. (2014). Do we Need Hundreds of Classifiers to Solve Real World Classification Problems? *Journal of Machine Learning Research*, *15*(1), 3133–3181.

Fisher, R. A. (1973). *Statistical methods for research workers* (14th ed.). New York, NY, USA: Hafner.

Froemelt, A., Dürrenmatt, D. J., & Hellweg, S. (2018). Using Data Mining To Assess

Environmental Impacts of Household Consumption Behaviors. *Environmental Science & Technology*, *52*(15), 8467–8478. https://doi.org/10.1021/acs.est.8b01452

Genuer, R., Poggi, J.-M., & Tuleau, C. (2008). *Random Forests: some methodological insights*. Retrieved from http://arxiv.org/abs/0811.3619

Hirschberg (ed.), S., Bauer, C., Cox, B., Heck, T., Hofer, J., Schenler, W., … Saner, D. (2016). *Opportunities and challenges for electric mobility: An interdisciplinary assessment of passenger vehicles*. Villigen, Dübendorf and Zurich, Switzerland.

Horni, A., Nagel, K., & Axhausen, K. W. (2016). *The Multi-Agent Transport Simulation Title of Book : The Multi-Agent Transport Simulation MATSim Subtitle positioned below*. https://doi.org/http://dx.doi.org/10.5334/baw

Hunter, J. D. (2007). Matplotlib: A 2D Graphics Environment. *Computing in Science and Engineering*, *9*, 90–95. https://doi.org/10.1109/MCSE.2007.55

Jones, E., Oliphant, T., Peterson, P., & et al. (n.d.). SciPy: Open Source Scientific Tools for Python. Retrieved April 1, 2017, from http://www.scipy.org/

Koch, P., & Salou, T. (2015). *AGRIBALYSE: Methodology Report - Version 1.2*. ADEME: Angers, France.

Kuhn, M., & Johnson, K. (2013). *Applied Predictive Modeling*. https://doi.org/10.1007/978-1-4614-6849-3

Liaw, A., & Wiener, M. (2002). Classification and Regression by randomForest. *R News*, *2*(3), 18–22. https://doi.org/10.1177/154405910408300516

McKinney, W. (2010). Data Structures for Statistical Computing in Python. *Proceedings of the 9th Python in Science Conference*, 51–56. Retrieved from http://conference.scipy.org/proceedings/scipy2010/mckinney.html

Meister, K., Balmer, M., Ciari, F., Horni, A., Rieser, M., Waraich, R. A., & Axhausen, K. W. (2010). Large-Scale Agent-Based Travel Demand Optimization Applied to Switzerland, Including Mode Choice. *12th World Conference on Transportation Research*. Lisbon, Portugal.

Mutel, C. (2017). Brightway: An open source framework for Life Cycle Assessment. *Journal of Open Source Software*, *12*, 2. https://doi.org/10.1021/es3050949.Mutel

Niculescu-Mizil, A., & Caruana, R. (2005). Predicting good probabilities with supervised learning. *Proceedings of the 22nd International Conference on Machine Learning - ACM*, 625–632. https://doi.org/10.1145/1102351.1102430

OECD. (2019). What are Equivalence Scales? Retrieved April 4, 2019, from http://www.oecd.org/els/soc/OECD-Note-EquivalenceScales.pdf

Pedregosa, F., Varoquaux, G., Gramfort, A., Michel, V., Thirion, B., Grisel, O., … Duchesnay, É. (2012). Scikit-learn: Machine Learning in Python. *Journal of Machine Learning Research*, *12*, 2825–2830. https://doi.org/10.1007/s13398-014-0173-7.2

Python Software Foundation. (2017). Python. Retrieved April 1, 2017, from https://www.python.org/

Saner, D., Heeren, N., Jäggi, B., Waraich, R. A., & Hellweg, S. (2013). Housing and Mobility Demands of Individual Households and their Life Cycle Assessment. *Environmental Science & Technology*, *47*(11), 5988–5997. https://doi.org/10.1021/es304084p

Schweizerischer Verein des Gas- und Wasserfaches (SVGW). (2017). Trinkwasser. Retrieved November 28, 2017, from http://wasserqualitaet.svgw.ch/index.php?id=874

The PostgreSQL Global Development Group. (n.d.). PostgreSQL. Retrieved January 19, 2017, from https://www.postgresql.org

Tukker, A., de Koning, A., Wood, R., Hawkins, T., Lutter, S., Acosta, J., … Kuenen, J. (2013). Exiopol - Development and Illustrative Analyses of a Detailed Global MR EE SUT/IOT. *Economic Systems Research*, *25*(1), 50–70. https://doi.org/10.1080/09535314.2012.761952

United Nations Statistics Division. (2017). COICOP (Classification of Individual Consumption According to Purpose). Retrieved November 22, 2017, from https://unstats.un.org/unsd/cr/registry/regcst.asp?Cl=5

Van Der Walt, S., Colbert, S. C., & Varoquaux, G. (2011). The NumPy Array: A Structure for Efficient Numerical Computation. *Computing in Science and Engineering*, *13*, 22–30. https://doi.org/10.1109/MCSE.2011.37

Varrazzo, D. (2017). psycopg. Retrieved April 1, 2017, from http://initd.org/psycopg/articles/

Waskom, M., Botvinnik, O., O'Kane, D., Hobson, P., Lukauskas, S., Gemperline, D. C., … Qualieh, A. (2017). Seaborn: Statistical Data Visualization. https://doi.org/10.5281/zenodo.883859

Wernet, G., Bauer, C., Steubing, B., Reinhard, J., Moreno-Ruiz, E., & Weidema, B. (2016). The ecoinvent database version 3 (part I): overview and methodology. *The International Journal of Life Cycle Assessment*, *21*(9), 1218–1230. https://doi.org/10.1007/s11367-016-1087-8

Wood, R., Stadler, K., Bulavskaya, T., Lutter, S., Giljum, S., de Koning, A., … Tukker, A. (2015). Global Sustainability Accounting-Developing EXIOBASE for Multi-Regional Footprint Analysis. *Sustainability (Switzerland)*, *7*(1), 138–163. https://doi.org/10.3390/su7010138
